# Locomotion dependent neuron-glia interactions control neurogenesis and regeneration in the adult zebrafish spinal cord

**DOI:** 10.1038/s41467-021-25052-1

**Published:** 2021-08-11

**Authors:** Weipang Chang, Andrea Pedroni, Maria Bertuzzi, Caghan Kizil, András Simon, Konstantinos Ampatzis

**Affiliations:** 1 Department of Neuroscience, Karolinska Institutet, Stockholm, Sweden; 2 German Center for Neurodegenerative Diseases (DZNE) within Helmholtz Association, Dresden, Germany; 3 Department of Neurology and the Taub Institute for Research on Alzheimer’s Disease and the Aging Brain, Columbia University Irving Medical Center, New York, NY USA; 4 Department of Cell and Molecular Biology, Karolinska Institutet, Stockholm, Sweden

**Keywords:** Spinal cord, Neural circuits, Adult neurogenesis, Spinal cord injury

## Abstract

Physical exercise stimulates adult neurogenesis, yet the underlying mechanisms remain poorly understood. A fundamental component of the innate neuroregenerative capacity of zebrafish is the proliferative and neurogenic ability of the neural stem/progenitor cells. Here, we show that in the intact spinal cord, this plasticity response can be activated by physical exercise by demonstrating that the cholinergic neurotransmission from spinal locomotor neurons activates spinal neural stem/progenitor cells, leading to neurogenesis in the adult zebrafish. We also show that GABA acts in a non-synaptic fashion to maintain neural stem/progenitor cell quiescence in the spinal cord and that training-induced activation of neurogenesis requires a reduction of GABA_A_ receptors. Furthermore, both pharmacological stimulation of cholinergic receptors, as well as interference with GABAergic signaling, promote functional recovery after spinal cord injury. Our findings provide a model for locomotor networks’ activity-dependent neurogenesis during homeostasis and regeneration in the adult zebrafish spinal cord.

## Abstract (web summary)

The mechanisms stimulating adult neurogenesis are unclear. Here, the authors show the contribution of cholinergic and GABAergic signalling within the locomotor network to spinal cord neurogenesis during homeostasis and regeneration, showing neurogenesis depends on circuit activity in the adult zebrafish.

## Introduction

Neurotransmitter signaling is traditionally associated with communication between neurons. However, several reports suggest that neurotransmitters also influence critical aspects of neurogenesis, including proliferation, migration, and differentiation, under both physiological and pathological conditions^1–12^. The association between neurotransmitter signaling and neurogenesis appears to be primarily dependent on transmitter receptors that are not confined to neurons. Such receptors are now known to be expressed on diverse cell types in the central nervous system, including stem and progenitor cells^4,13^. Therefore, neuronal network activity can directly affect neurogenesis^8,14^. Previous studies highlighted a link between neurogenesis and neurotransmission, showing the direct effects of the cholinergic and GABAergic signaling in the modulation of the stem/progenitor cells in the mammalian hippocampus and spinal cord^3,8,13,15–17^, yet it remains unclear how neuronal activity is linked to neurogenic activity in the adult spinal cord. Hence, we hypothesized that prolonged spinal network activity, after training, could stimulate the animal growth rate by engaging the spinal proliferative and neurogenic programs.

In the early development of the vertebrate spinal cord, all neurons follow a specific genetic program that defines their identities and assigns them a specific neurotransmitter phenotype^18^. Spinal neurons are organized into distinct networks that integrate and process sensory and motor-related information important for various movements^19–21^. Among the spinal networks, the central pattern generators (CPGs) function as local “control and command” centers that are essential for generating the rhythmicity and coordination required for muscle activity during locomotion^19–21^. At the level of spinal locomotor circuits, several classes of premotor interneurons use specific neurotransmitters, including glutamate, γ-aminobutyric acid (GABA), glycine, and acetylcholine (ACh), to mediate their functions^22^. However, it is unknown whether these neurotransmitters released during locomotion can directly affect the neural stem/progenitor cells (NSPCs) within the spinal cord. If so, by identifying neurotransmitters with neurogenic potential could expose the neurons that control these processes. Therefore, neurotransmitter signaling may play an essential activity-dependent role in regulating and fine-tuning the adult spinal cord neurogenesis.

To determine whether physical activity can induce spinal cord neurogenesis, we applied an array of anatomical, pharmacological, electrophysiological, and behavioral approaches in adult zebrafish. Our data demonstrate that cholinergic (synaptic) and GABAergic (non-synaptic) neurotransmission regulates the activity of the NSPCs in opposite manners. We show that among spinal interneurons, it is the locomotor V2a interneurons that mediate the essential cholinergic input to NSPCs. The results demonstrate that spinal network activity plays a crucial role in modulating non-motor and non-neuronal functions in the nervous system besides generating motor behaviors.

## Results

### Physical activity induces animal growth and proliferation in the spinal cord

**Fig. 1:**
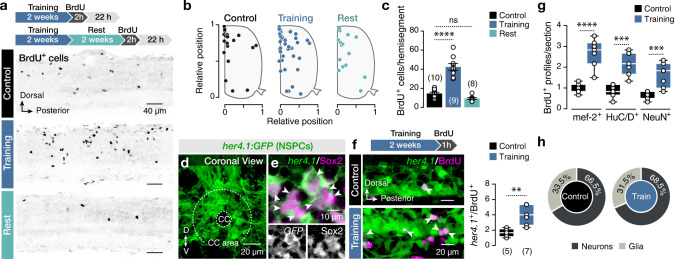
Exercise-induced transient activation of the NSPCs and neurogenesis in the adult spinal cord. **a** Inverted confocal images from whole-mount adult zebrafish spinal cord hemisegments showing cycling (BrdU^+^) cells in control animals (untrained), following 2 weeks of training and 2 weeks rest after training. **b** Similar distribution pattern of BrdU^+^ cells in the spinal cord comparing untrained, trained, and resting zebrafish. **c** Quantification of BrdU^+^ cells per hemisegment in different conditions show that the enhanced proliferation after training is reversible (*P* = 4.418E-10). **d** Expression pattern of *her4.1*:*GFP* (NSPCs; green) in close apposition of the adult zebrafish spinal cord’s central canal. **e** The vast majority (~97.5%) of the *her4.1*^+^ cells (green) express the stem cell marker Sox2 (magenta). Arrowheads indicate double-labeled cells. **f** Cycling *her4.1*^+^ radial glia cells (BrdU^+^, magenta; GFP, green). Training increased the number of BrdU^+^/*her4.1*^+^ cells per hemisegment. **g** Quantification of the average BrdU^+^ cells per spinal cord section co-expressing neuronal markers (mef-2, HuC/D, or NeuN) in untrained (control) and trained animals. **h** Proportions of BrdU^+^ cells expressing neuronal or glial markers are similar comparing untrained and trained animals. Quantification is based on the early neuronal marker mef-2. BrdU, 5-bromo-2ʹ-deoxyuridine; CC, central canal; GFP, green fluorescent protein; her4.1, hairy-related 4, tandem duplicate 1; HuC/D, elav3 + 4; mef-2, myocyte enhancer factor-2; NeuN, neuronal nuclei; NSPC, neural stem/progenitor cell; Sox2, sex-determining region Y-box 2. Data are presented as mean ± s.e.m. or as box plots showing the median with 25/75 percentile (box and line) and minimum–maximum (whiskers). ^**^P < 0.01; ^***^P < 0.001; ^****^P < 0.0001; ns, not significant. For detailed statistics, see Supplementary Table 1.

Several studies have documented the impact of physical activity on neurogenesis in the mammalian hippocampus^11,23–26^. Unlike mammals, zebrafish retain a remarkable adult neurogenic capacity in many central nervous system areas, including the spinal cord^27–30^. We first tested whether physical activity leads to proliferative and neurogenic events and assayed global consequences on animal growth by using our recently developed forced swim protocol^31^. We observed that prolonged physical activity (>2 weeks) significantly increased animal growth (Supplementary Fig. 1). Combining our exercise protocol with the thymidine analog 5-bromo-2ʹ-deoxyuridine (BrdU), a marker of DNA synthesis, we observed a 3-fold increase in the number of BrdU^+^ cells in the spinal cord after 2 weeks of training (short-term survival; Fig. 1a–c). After a BrdU pulse, we could also trace the migrated cells out of the proliferative central canal niche (Fig. 1a, b). After 2 weeks rest from the exercise, the proliferation rate dropped to the level of untrained control animals (Fig. 1a, c), demonstrating the dynamic and reversible nature of exercise-induced proliferation.

### NSPCs in the adult spinal cord respond by increased proliferative activity to physical training

Specialized glial cells are lining the spinal cord’s central canal, a proliferative niche harboring NSPCs in both fish and mammals^32–36^. In zebrafish, the calcium-binding protein calbindin (CB) selectively marks cells surrounding the spinal cord’s central canal (Supplementary Fig. 2a)^37^. Moreover, CB is extensively colocalized with the stem cell marker Sox2 (Supplementary Fig. 2b) but is not expressed in the GABAergic cerebrospinal fluid contacting neurons (CSF-cNs; Supplementary Fig. 2c) adjacent to the central canal^38^. To corroborate that CB selectively marks spinal NSPCs, we used the *her4*.*1:GFP* transgenic reporter line that marks NSPCs in the zebrafish CNS (Fig. 1d and Supplementary Fig. 2d)^39–41^. We found that none of the radial glia-like GFP^+^ cells expressed the neuronal marker HuC/D (Supplementary Fig. 2e; HuC/D^−^), all GFP^+^ were CB^+^ (Supplementary Fig. 2f), and that the vast majority of GFP^+^ cells were also expressing Sox2 (Fig. 1e). Double labeling after BrdU pulse during physical training (Fig. 1f) showed an increased number of *her4.1:GFP*^+^BrdU^+^ cells after training (Fig. 1f), indicating increased NSPCs’ proliferation in response to physical activity.

### Most newborn cells differentiate into neurons after physical activity

Next, we sought to examine the fate of new cells in the adult spinal cord 2 weeks after BrdU treatment (Supplementary Fig. 3). A majority (~68%) of the BrdU^+^ cells expressed either the early differentiation neuronal marker mef-2 or the post-mitotic pan-neuronal markers HuC/D and NeuN (Fig. 1g, h). In contrast, a small fraction (~32%) of BrdU^+^ cells expressed the glial marker GFAP (Supplementary Fig. 3a, b). In both control and trained animals, the proportion of newborn cells expressing glial versus neuronal markers remained unaltered, suggesting that physical activity did not affect newborn cells’ differentiation fate (Fig. 1h and Supplementary Fig. 3b).

### NSPCs receive neuronal input during locomotion

**Fig. 2:**
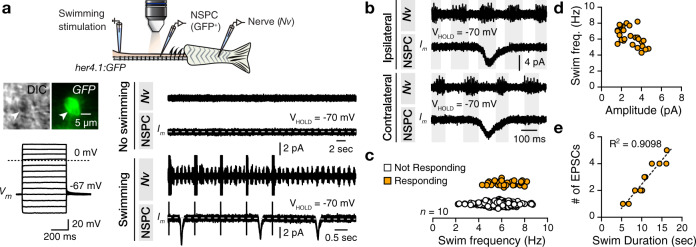
NSPCs receive periodic input from the locomotor network. **a** Images of a NSPC (*her4.1:GFP*^+^, white arrowhead) close to the spinal cord’s central canal. Current steps do not produce action potentials in NSPCs. Ex vivo setup of the brain-spinal cord preparation allows simultaneous recordings of a spinal cord NSPC and ipsilateral motor nerves (*Nv*). Electrical stimulation (10 pulses at 1 Hz) of the descending inputs elicits a swimming episode. In the absence of swimming, NSPCs do not respond (top traces). During a fictive locomotor episode, NSPCs periodically receive strong inputs (bottom traces). **b** NSPC responses during swim phase and the locomotor cycle during simultaneous ipsilateral and contralateral recordings. **c** Graph showing the activity of different NSPCs as a function of instantaneous swimming burst frequency. Individual data points represent instantaneous swimming frequencies of all swimming cycles where the respective NSPCs responded. **d** No apparent correlation between the amplitude of the periodic NSPC responses and the swimming frequency. **e** Correlation (*R*^2^ = 0.9098) between the swim duration and the detected number of inputs to NSPCs. The dashed gray line represents the baseline. NSPC, neural stem/progenitor cell. For detailed statistics, see Supplementary Table 1.

Next, we examined whether the spinal locomotor network is directly implicated in the activation of the NSPCs. We performed whole-cell patch-clamp recordings in single NSPCs while recording motor nerve activity of the ipsilateral CPG in ex vivo adult *her4.1:GFP* zebrafish preparation^42,43^ (Fig. 2a). We verified that the GFP^+^ cells had glial physiological properties, such as hyperpolarized resting membrane potential (−68.27 ± 0.7 mV), linear voltage–current relations, and no generation of action potentials (Fig. 2a and Supplementary Fig. 4). Moreover, in the absence of the electrically induced fictive swimming, the NSPCs did not receive any synaptic input (Fig. 2a). However, after initiating fictive locomotion by electric stimulation (10 pulses, 1 Hz) of the descending axons from the brainstem, we detected a strong periodic synaptic input in NSPCs at frequencies above 4 Hz (Fig. 2a, c). Moreover, this input was always in phase to the CPG activity (Fig. 2b). To further confirm that the NSPCs’ inputs were causally associated with the CPG activity, we also recorded from contralaterally located NSPCs and found that they displayed out-of-phase relations (Fig. 2b). This differential phase-locked association between the CPG activity and the NSPC input suggested that this was a locomotor network’s activity outcome. Swimming burst frequency and strength showed no correlation to the NSPCs’ response (Fig. 2d). Nevertheless, the locomotor episodes’ duration correlated with the periodic NSPCs’ response (Fig. 2e). Together, these data link locomotor network and NSPCs’ activity but reveal neither the nature of this input nor the spinal locomotor interneurons involved.

### NSPCs receive synaptic cholinergic input and non-synaptic GABAergic input

**Fig. 3:**
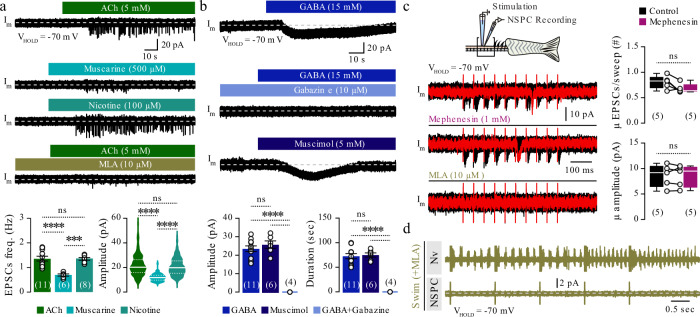
NSPCs respond to synaptic cholinergic input from the locomotor network and to non-synaptic GABAergic signaling. **a** Bath application of ACh induced inward currents in all recorded NSPCs (20 out of 20). Sample traces of the muscarine- and nicotine-induced inward currents in recorded NSPCs. Significant reduction of the induced ACh currents in the presence of the α7 nicotinic receptor antagonist MLA (10 μM). Quantification of the frequency (Hz; *P* = 3.237E-5) and amplitude (pA; *P* < 0.0001) of the recorded cholinergic currents. **b** Exogenous application of GABA induced tonic activation of NSPCs (22 out of 22). GABAergic tonic responses were completely abolished in the presence of the GABA_A_ receptor antagonist, gabazine (10 μΜ). Exogenous application of muscimol (GABA_A_ receptor agonist) induced tonic activation of NSPCs. Quantification of the amplitude (pA; *P* = 7.904E-8) and duration (s; *P* = 1.141E-7) of the GABA-related responses. **c** Schematic protocol for NSPC recordings during local electrical stimulation. Ten pulses (20 Hz) were applied to increase the probability of presynaptic release. Superimposed representative sample trace (in red) out of >40 sweeps (in black) from NSPC responses under control conditions, following application of the polysynaptic blocker mephenesin, and application of the selective nicotinic receptor antagonist MLA suggesting synaptic cholinergic, but not GABAergic activation of the NSPCs. Quantification of the average number of detected EPSCs per sweep and the average amplitude of the responses in control and after the application of polysynaptic blocker (mephenesin). **d** Application of MLA during locomotion abolishes the regular and strong input to NSPCs, implying a predominant role of nicotinic receptors. ACh, acetylcholine; GABA, γ-aminobutyric acid; MLA, methyllycaconitine; NSPC, neural stem/progenitor cell; *Nv*, motor nerve recording. The dashed gray line represents the baseline. Data are presented as mean ± s.e.m. and as violin plots. ^***^P < 0.001; ^****^P < 0.0001; ns, not significant. For detailed statistics, see Supplementary Table 1.

We performed whole-cell electrophysiological recordings from the NSPCs (*her4*.*1:GFP*^*+*^) upon stimulation with neurotransmitters using an intact ex vivo adult zebrafish spinal cord preparation. Among the tested neurotransmitters (glutamate, glycine, serotonin, ACh, and GABA; Supplementary Fig. 5a), only ACh and GABA induced noticeable changes in the NSPCs (Fig. 3a, b and Supplementary Fig. 5b–h). Bath application of ACh (100 μM or 5 mΜ) induced numerous excitatory postsynaptic currents (EPSCs) with variable amplitudes in a dose-dependent manner (Supplementary Fig. 5b, c). The action potential blocker tetrodotoxin (TTX) affected neither the frequency nor the amplitude of the EPSCs, confirming the presence of ACh receptors on the NSPCs’ membrane (Supplementary Fig. 5b, c). Next, we aimed to distinguish between muscarinic and nicotinic-ACh receptors (Fig. 3a) in this membrane-associated activity. Activation of muscarinic (muscarine, 500 μM) and nicotinic (nicotine, 100 μM) ACh receptors generated EPSCs with different frequencies and amplitudes (Fig. 3a). Stimulation of nicotinic receptors better recapitulated the results induced by ACh compared to stimulation of muscarinic receptors, indicating that the cholinergic input on the NSPCs is predominantly mediated by nicotinic receptors (Fig. 3a). Treatment with the selective α7 nicotinic-ACh receptor antagonist methyllycaconitine (MLA, 10 μM) significantly reduced the recorded EPSCs (Fig. 3a), further supporting the central role of nicotinic receptors. Next, we assessed the GABAergic responses to NSPCs (Fig. 3b and Supplementary Fig. 5d, f, h). NSPCs responded to bath application of the neurotransmitter GABA by inducing a prominent inward tonic activation (depolarization; Fig. 3b and Supplementary Fig. 5h) insensitive to TTX (Supplementary Fig. 5d). These GABA-mediated tonic responses were blocked entirely by the GABA_A_ receptor antagonist gabazine (10 μM; Fig. 3b). Applying the selective GABA_A_ receptor agonist muscimol (15 mM; Fig. 3b) could also accurately generate tonic activation.

Next, we examined the nature of the cholinergic and GABAergic transmission to NSPCs. We applied electrical stimulation of the spinal cord to depolarize all neurons, thereby increasing neurotransmitter release (Fig. 3c). Following the electrical stimulation, we observed that the NSPCs received solely monosynaptic cholinergic inputs, as they were unaffected (number and amplitude of recorded EPSCs) by the presence of the polysynaptic blocker mephenesin (Fig. 3c), while activity was entirely blocked by the selective α7 nicotinic-ACh receptor antagonist MLA (Fig. 3c). These data suggest that the GABAergic signaling to NSPCs is non-synaptic, as previously found in the mammalian brain^3,8,44^. Furthermore, we observed that MLA abolished the regular strong input to the NSPCs during fictive locomotion, indicating that locomotion-induced signal is solely cholinergic and mediated through the α7 nicotinic receptors (Fig. 3d).

### Premotor V2a interneurons mediate the essential cholinergic input to NSPCs during locomotion

**Fig. 4:**
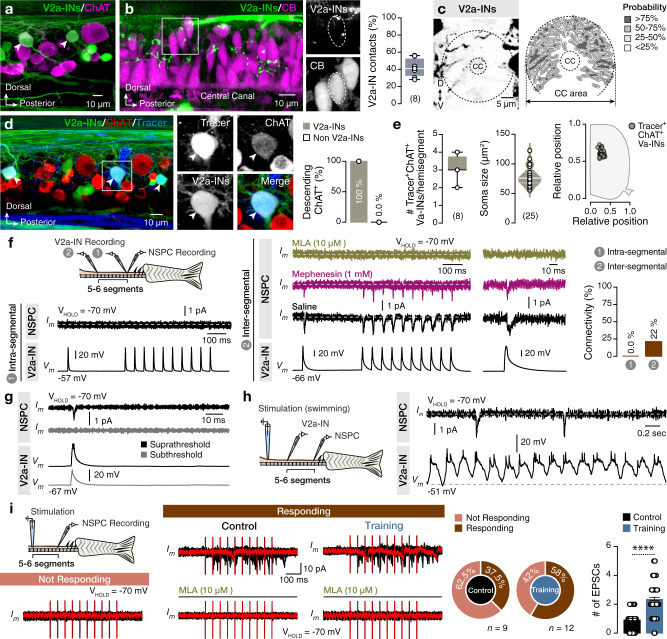
Spinal locomotor V2a-INs contribute cholinergic inputs to NSPCs. **a** Large and dorsally located spinal cord V2a-INs (*Chx10:GFP*^*+*^, green) are cholinergic (ChAT^+^, magenta). Arrowheads indicate double-labeled neurons (*Chx10:GFP*^*+*^ChAT^+^). **b** A sample stack from the central canal area showing the presence of V2a-IN (*Chx10:GFP*^*+*^) axonal collaterals (green) close to CB^+^ NSPCs (magenta) with analysis of the proportion of the CB^+^ NSPCs that are in close proximity with the V2a-IN (*GFP*^*+*^) processes. **c** Quantification of the probability of the V2a-IN axonal collaterals in the central canal region (*n* = 15 zebrafish). **d** Representative whole-mount confocal image showing that all (25 out of 25; 100% from 8 zebrafish) long descending (dextran tracer, blue; >10 segments) cholinergic (ChAT^+^, red) neurons are V2a-INs (GFP^+^, green). Arrowheads indicate triple-labeled neurons. **e** Quantification and analysis of the number, size and location of long descending cholinergic V2a-INs in the adult zebrafish spinal hemisegments. **f** Sample average (~25 sweeps) traces from dual electrophysiological recordings between a premotor V2a-IN and NSPCs, located in the same segment (intra-segmental, 1) or 5–6 segments rostrally (inter-segmental, 2). Cholinergic connections were observed in the inter-segmental pairs (22%, 11 out of 50 pairs) but not in the intra-segmental pairs (0%, 0 out of 15). **g** Postsynaptic responses in the recorder NSPCs generated from suprathreshold (black, with action potential) and not from subthreshold (gray, without an action potential) short pulse depolarization of the V2a-IN. **h** Ex vivo setup of the brain-spinal cord preparation allows simultaneous recordings of a NSPC and ipsilateral descending V2a-INs during fictive locomotion. Sample trace of a connected pair that, while the V2a-IN discharges during fictive swimming, the NSPC receives occasional input. **i** Illustrat**i**on of recordings acquired during electrical stimulations. Ten pulses (20 Hz) of a rostral spinal cord segment were applied to depolarize V2a-INs connected to NSCs. Representative sample trace (in red) of superimposed sweeps (~20, in black) from not responding and responding NSPCs. Bath application of the selective nicotinic antagonist MLA (10 μM) abolished the recorded currents suggesting that they are cholinergic. Changes in the proportion of the recorded NSPCs that respond to electrical stimulation observed after training (*n*: number of recorded NSPCs). The average number of detected events per stimulation sweep from both sites was significantly higher in trained animals, suggesting adaptive changes in the innervation and the cholinergic release to the NSCs (*P* < 0.0001). The dashed gray line represents the baseline. CB, calbindin D-28K; CC, central canal; ChAT, choline acetyltransferase; EPSC, excitatory postsynaptic current; INs, interneurons; MLA, methyllycaconitine; NSPC, neural stem/progenitor cell. Data are presented as mean ± s.e.m., as violin plots and as box plots showing the median with 25/75 percentile (box and line) and minimum–maximum (whiskers). ^****^P < 0.0001. For detailed statistics, see Supplementary Table 1.

To gain insight into the ACh’s potential sources to NSPCs, we focused on spinal cholinergic interneurons^45^. Cholinergic V2a interneurons (INs)^22^ (Fig. 4a) are the principal components of the locomotor CPG^19–21,43,46,47^. Anatomical analysis revealed close appositions of V2a-IN axonal collaterals (*Chx10:GFP*^+^) to the central canal area (Fig. 4b, c) while we identified that ~40% of the NSPCs (CB^+^) had close proximities, likely synaptic contacts, with the V2a-INs (GFP^+^; Fig. 4b). To functionally test whether V2a-INs could provide cholinergic input to NSPCs, we performed pair recordings from the same segment (Fig. 4f). We observed that a train of action potentials elicited in V2a-INs failed to induce any postsynaptic responses to NSPCs (intra-segmental: 0 out of 15 pairs; Fig. 4f). However, V2a-INs are long ipsilateral descending neurons, and we observed that all (25 out of the 25; *n* = 8 zebrafish) long descending (>10 segments) spinal cholinergic neurons in zebrafish were indeed V2a-INs (GFP^+^; Fig. 4d). Moreover, we identified that the descending cholinergic V2a-INs (~3/hemisegment) have medium-to-large body size and specific dorsomedial location in the spinal cord (Fig. 4e). Pair recordings obtained from distal segments (~5–7 segments apart; inter-segmental) revealed that action potentials in V2a-INs induced vigorous small-amplitude EPSCs in NSPCs in 22% of the cases (inter-segmental: 11 out of 50 pairs; Fig. 4f, g). The recorded EPSCs were resistant to mephenesin (1 mM), a pharmacological agent shown to act as a potential polysynaptic transmission blocker in the mammalian spinal cord^48^ (Fig. 4f and Supplementary Fig. 6). Yet, the observed changes in the duration of the recorded EPSCs after the application of mephenesin suggested that the interaction between V2a-INs and NSPCs comprises monosynaptic and polysynaptic inputs (Supplementary Fig. 6). To further determine whether these responses are cholinergic, we applied the nicotinic receptor antagonist MLA and observed that it completely abolished the monosynaptic EPSCs in NSPCs (Fig. 4f). We noticed that the transmission between V2a-INs and NSPCs exhibited partial and complete failures in ~20% of the cases during train stimulation (Supplementary Fig. 7), suggesting that nicotinic-ACh receptors might undergo desensitization, which is a potential mechanism to control synaptic efficacy^49,50^. Next, we asked whether V2a-INs release cholinergic input to NSPCs during locomotion. Simultaneous recordings from connected pairs of V2a-INs and NSPCs revealed that while V2a-INs discharged rhythmically during swimming, NSPCs occasionally receive this cholinergic input (Fig. 4h) as seen before (Fig. 2a), implying that the nicotinic-ACh receptor desensitization most likely is responsible for the non-regular release of the ACh during locomotion.

Finally, we tested whether NSPCs adapt their response to signals from rostral segments (segments 9–11) during training (Fig. 4i). We noticed that training caused an increase in recruiting NSPCs that receive a nicotine-mediated synaptic input from descending V2a-INs by 18% (Fig. 4i). Moreover, we found that the number of detected EPSC events per stimulation sweep increased by ~150% (Fig. 4i). These data suggest that physical activity increased the cholinergic release to NSPCs and expanded V2a-IN cholinergic synapses to NSPCs. However, their extensive and complex branched morphology precluded a strict quantification of this structural plasticity.

### Cholinergic and GABAergic receptors control the NSPCs’ proliferation in an opposing manner

**Fig. 5:**
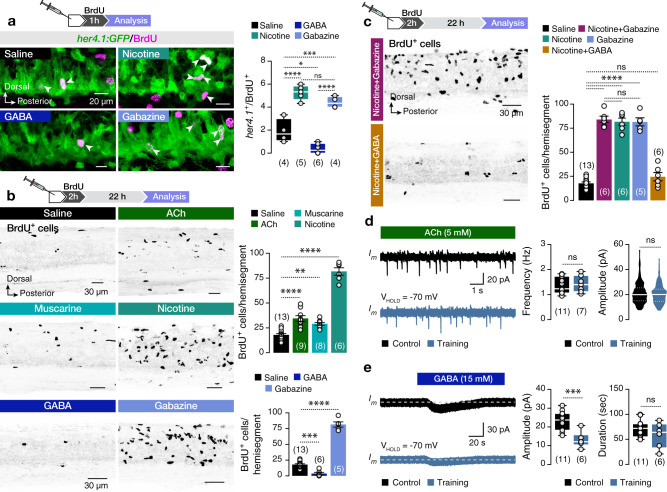
Cholinergic and GABAergic receptors control the NSPCs’ proliferation in an opposing manner. **a** Microphotographs and analysis show that nicotine and gabazine increased the *her4.1*^+^ (green)/BrdU^+^ (magenta) cells, whereas GABA reduced the number of *her4.1*^+^/BrdU^+^ cells in the examined spinal hemisegment (*P* < 0.0001). **b** In vivo administration of ACh, muscarine, nicotine, and gabazine increased the number of BrdU^+^ cells per hemisegment (*P* < 0.0001). Administration of GABA reduced the number of BrdU^+^ cells in the adult zebrafish spinal cord hemisegment (*P* < 0.0001). **c** Co-administration of nicotine and gabazine generated the same number of BrdU^+^ cells as the individual administration of nicotine or gabazine. Co-activation of the nicotinic-ACh receptors and the GABA_A_ receptors produced the same number of BrdU^+^ cells as in control (saline; *P* < 0.0001). **d** Application of ACh induced currents of the same frequency and amplitude in NSPCs before and after training, suggesting no changes in the cholinergic receptors following training. **e** Bath application of GABA before and after training revealed a significant reduction of the tonic activation amplitude without affecting its duration in the NSPCs. ACh, acetylcholine; BrdU, 5-bromo-2ʹ-deoxyuridine; GABA, γ-aminobutyric acid; GFP, green fluorescent protein; her4.1, hairy-related 4, tandem duplicate 1; The dashed gray line represents the baseline. Data are presented as mean ± s.e.m., as violin plots and as box plots showing the median with 25/75 percentile (box and line) and minimum–maximum (whiskers). *P < 0.05; **P < 0.01; ***P < 0.001; ****P < 0.0001; ns, not significant. For detailed statistics, see Supplementary Table 1.

To test whether manipulation of nicotinic-ACh and GABA_A_ receptors impinges NSPCs’ proliferation, *her4.1*^+^BrdU^+^ cells lining the central canal were quantified after a single administration of nicotine, GABA, or gabazine followed by a pulse with BrdU for 1 h (Fig. 5a). We observed a significant increase in the number of *her4.1*^+^BrdU^+^ cells after exposure to nicotine and gabazine (Fig. 5a). In contrast, GABA_A_ receptor activation by ambient GABA significantly reduced the number of BrdU *her4.1*^+^BrdU^+^ cells (Fig. 5a).

We next examined how cholinergic activation of NSPCs engenders the proliferation in vivo by injecting adult zebrafish intraperitoneally with ACh (2 mM), muscarine (a selective agonist of muscarinic-ACh receptors, 50 μM), or nicotine (a selective agonist of nicotinic-ACh receptors, 300 μM) (Fig. 5b). Activation of the ACh receptors significantly increased the number of BrdU^+^ cells in the spinal cord (Fig. 5b). Notably, nicotine administration resulted in a higher number of BrdU^+^ cells than ACh, reflecting that extracellular acetylcholinesterases rapidly degrade ACh. In contrast, GABA (500 μM) or the GABA_A_ receptor antagonist gabazine (100 μM; Fig. 5b) caused a decrease in the number of BrdU^+^ cells. Conversely, the number of BrdU^+^ cells increased in animals treated with the GABA antagonist gabazine (Fig. 5b). To verify that neurotransmitters acted directly on spinal cord receptors and not through a systemic effect, we treated isolated intact cords with cholinergic and GABAergic antagonists and agonists ex vivo (Supplementary Fig. 8a). In accordance with the in vivo studies, we observed a significant increase in proliferation upon selective activation of cholinergic receptors (Supplementary Fig. 8b). In contrast, GABA-treated adult spinal cords showed a significant decrease in the detected BrdU^+^ cells, and gabazine caused a significant increase in proliferation (Supplementary Fig. 8b). We also treated the isolated intact spinal cords with nicotine, GABA, and gabazine in the presence of the synaptic blocker TTX (Supplementary Fig. 9a). We observed that activation of the nicotinic receptors and blockage of the GABA_A_ receptors (gabazine) enhanced the proliferation (BrdU^+^ cells), while GABA reduced the number of the newborn cells (Supplementary Fig. 9b). Collectively, our data suggest that the observed changes in the proliferation resulted from the direct modulation of neurotransmitter receptors on spinal cord NSPCs.

Next, we determined how simultaneous manipulation of nicotinic-Ach and GABA_A_ receptors influenced proliferation. We found that activation of the nicotinic-ACh receptors along with the blockage of the GABA_A_ receptors by co-administration of nicotine and gabazine did not produce more BrdU^+^ cells than each manipulation separately (Fig. 5c). When we performed nicotine and GABA co-injections, we observed that the number of the BrdU^+^ cells was the same as in control (saline-injected) animals (Fig. 5c). These results collectively show that direct activation of nicotinic receptors triggers NSPCs and induces the proliferative program that is counteracted by GABA_A_ receptors.

### Adaptive regulation of NSPC GABA_A_ receptors after training

We determined whether physical training results in changes in NSPC receptors. We observed no changes in the frequency or amplitude of ACh-induced EPSCs in the NSPCs upon training (Fig. 5d), suggesting that the number of cholinergic receptors remained unaltered. On the contrary, the responses observed by the NSPCs from trained animals after treatment with GABA (15 mM) had significantly lower amplitude than those observed in control (untrained) animals (Fig. 5e). However, their duration was unaffected (Fig. 5e), implying a reduction in GABA_A_ receptors’ abundance after prolonged physical activity. These findings collectively suggest that two distinct and complementary mechanisms regulate spinal cord proliferation after training: an increase in cholinergic neurotransmission (a synaptic/network mechanism) and a reduction in the number of GABA_A_ receptors (a self-regulatory mechanism) in the NSPCs.

### Manipulation of neurotransmitter receptors on NSPCs promotes neuronal regeneration and restoration of motor functions

**Fig. 6:**
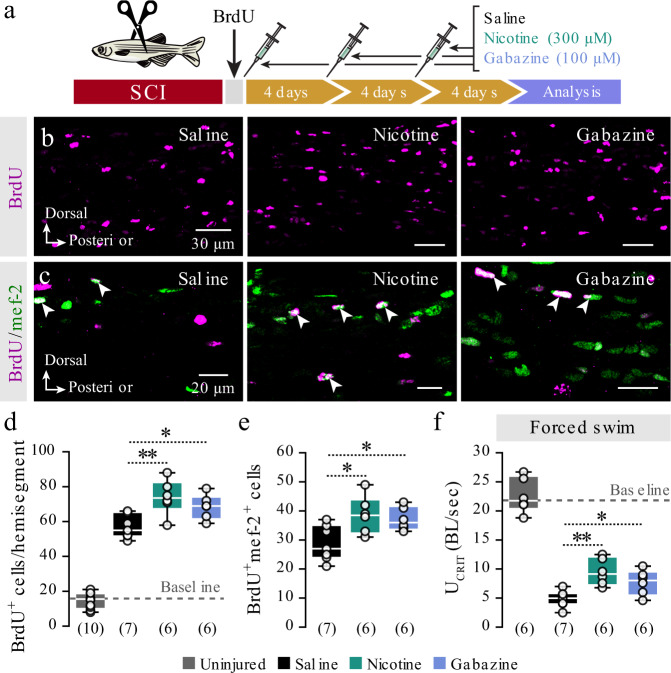
Nicotine and gabazine promote neurogenesis and restoration of motor performance after spinal cord injury. **a** Pulse-chase experiment to assess proliferation, neurogenesis, and restoration of motor functions after pharmacological manipulation of the nicotinic-ACh and GABA_A_ receptors in the adult zebrafish spinal cord. **b** Representative whole-mount confocal microphotographs showing BrdU^+^ cells in the zebrafish spinal cord. **c** Representative whole-mount confocal images for immunodetection of BrdU^+^/mef-2^+^ cells. Arrowheads indicate double-labeled cells. **d** Quantification of BrdU-incorporation after injury in control (saline) and pharmacologically treated animals (nicotine, gabazine). The dashed gray line represents the baseline (BrdU^+^ cells in uninjured animals). **e** Quantification of the BrdU^+^ cells express the neuronal marker mef-2^+^. **f** Nicotine- and gabazine-treated animals swim faster than the control (saline) fish during the critical speed test. The dashed gray line represents the baseline (critical speed of the uninjured animals). Speed is normalized (BL/s). BL, body length; BrdU, 5-bromo-2ʹ-deoxyuridine; mef-2, myocyte enhancer factor-2; SCI, spinal cord injury. Data are presented as box plots showing the median with 25/75 percentile (box and line) and minimum–maximum (whiskers). ^*^P < 0.05; ^**^P < 0.01. For detailed statistics, see Supplementary Table 1.

Spinal cord regeneration involves an extensive proliferation of NSPCs and subsequent neurogenesis. Indeed, following transection of the spinal cord at segment 15, we observed a significant increase in the number of BrdU^+^ cells (Fig. 6a). We asked whether pharmacological manipulation of nicotinic-ACh and GABA_A_ receptors could promote regeneration (Fig. 6). Animals that received pharmacological treatment with either nicotine or gabazine produced higher number of BrdU^+^ cells than the saline-treated fish (Fig. 6b, d). The increased proliferation correlated with increased neurogenesis assayed by BrdU^+^mef-2^+^ (Fig. 6c, e). These data suggest that pharmacological treatments can effectively bolster proliferation and neurogenesis in the injured animals. We found that both nicotine- and gabazine-treated fish recovered locomotion performance faster than the untreated animals after spinal cord transection, suggesting an accelerated regeneration process (Fig. 6f). Thus, spinal cord regeneration could be promoted by either increasing the cholinergic signaling or blocking the GABAergic signaling to NSPCs.

## Discussion

**Fig. 7:**
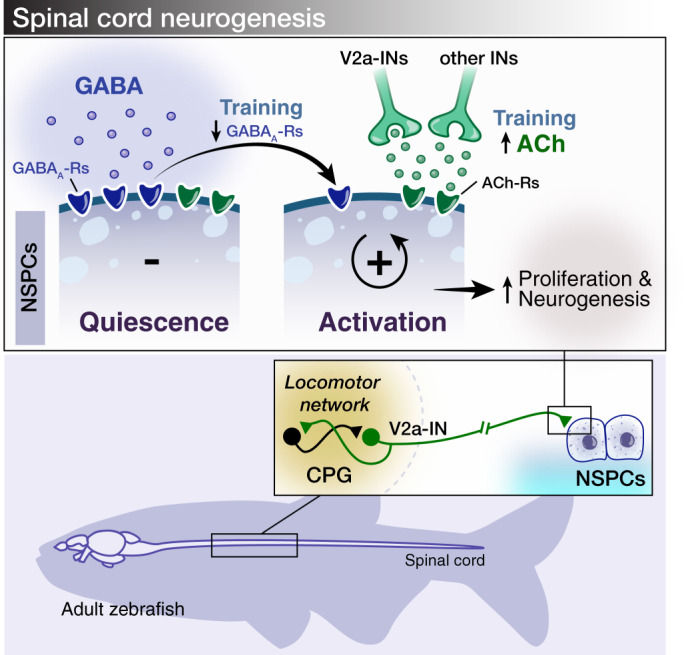
Proposed model for exercise controlled adult neurogenesis in the zebrafish spinal cord. The findings link the locomotor CPG network to adult neurogenesis. Spinal cholinergic interneurons, including the premotor V2a-IN population, increase their cholinergic release to NSPCs during training. ACh acts directly on the NSPCs via nicotinic and muscarinic cholinergic receptors to activate them. Activation of NSPCs leads to the downregulation of GABA_A_ receptors.

Here we revealed an adaptive mechanism by which physical activity dynamically modulates adult neurogenesis mediated by ACh and GABA neurotransmitters. Specifically, the locomotor CPG V2a-INs link motor functions to neurogenesis by contributing to regulation of the NSPCs’ proliferation and subsequent neurogenesis. While activation of NSPCs relies on an increased synaptic cholinergic input and is independent of the number of cholinergic receptors, insensitivity to non-synaptic GABAergic signaling^3,8^ is achieved by reducing the abundance of GABA_A_ receptors (Fig. 7). Therefore, exercise-dependent neurogenesis involves two distinct and mutually antagonistic processes. Extending these findings to a spinal cord injury model, we found that activation of the nicotinic-ACh receptors and inhibition of the GABA_A_ receptors increased the number of newborn neurons and promoted motor function restoration.

Previous studies have shown that several neurotransmitters can directly or indirectly regulate the activity of the NSPCs and neurogenesis in the nervous system^3,8–11,13,14,16,51–53^. Our results highlight the neurotransmitter ACh’s pivotal role in mediating physical activity-induced proliferation and neurogenesis. The evolutionarily conserved role of ACh and GABA in regulating mammalian hippocampal neurogenesis^3,8,16,17^ and spinal cord gliogenesis^13,15^, further indicates that these transmitters aim to control the generation of new cells without affecting their differentiation program. Indeed, it has been shown that after spinal cord injury, mammalian spinal cord progenitor/stem/progenitor cells exhibit a robust but abortive proliferative response that fails to generate mature neurons^54^, but rather produces the glial scar formation^33,55^. Conversely, zebrafish can fully regenerate the spinal cord and recover motor and sensory functions by activating the neurogenic program of the NSPCs around the central canal^34,56^. The antagonistic effects of ACh and GABA transmitter activity on NSPCs probably operate through a dynamic interplay with the molecular context provided by certain molecular factors, but the rules that govern this interaction still remain unclear.

We also investigated the role of other neurotransmitters in the direct control of NSPCs. While serotonin is known to regulate proliferation and neurogenesis in various contexts^10,41,52,57,58^, its precise mechanism of action has remained unexplored^59,60^. Our results indicate that serotonin, glutamate, and glycine^22^ are not directly involved in spinal NSPCs’ proliferation. In support, our previous study showed that serotonergic inputs modulate NSPCs’ proliferation in the brain indirectly through other signaling molecules, such as the brain-derived neurotrophic factor (BDNF)^41,61^. Similarly, glutamate has also been suggested to act on NSPCs indirectly, via modulation of neurotrophic factors, such as the BDNF and the nerve growth factor (NGF) and fibroblast growth factor (FGF)^10,62–65^.

It is conceivable, indeed probable, that adaptation occurs within the spinal cord after training^31^. The exact mechanisms of this adaptation are unclear, and the present data generate several testable hypotheses. For example, neurotransmitter switching is a recently discovered form of plasticity^66,67^ whereby neurons change their transmitter phenotypes in response to a sustained stimulus such as exercise^31^. As such, it is an activity-dependent adaptive mechanism^31,68^ that could explain changes in neurotransmitter availability and equilibrium that occur in the nervous system under both physiological as well as pathophysiological conditions. Further studies could examine the extent to which increased cholinergic neurotransmission to stem cells is mediated through cholinergic re-specification of the spinal interneurons.

The findings reported here show that the activity of the locomotor networks induces NSPCs to proliferate. We found that cholinergic V2a-INs are among the spinal interneurons interacting with the NSPCs, presumably using direct (monosynaptically) and indirect (polysynaptically) connectivity. We observed reliable, fast, and time-locked cholinergic reactions in the NSPCs triggered by V2a-IN spikes, a result that is commonly interpreted as reflecting direct monosynaptic input (Fig. 4f and Supplementary Fig. 6). Yet, the observed changes in the shape of the EPSCs after applying mephenesin also suggested the presence of a polysynaptic component (Supplementary Fig. 6). Even if a component of the observed action potential-mediated signals is transmitted through a downstream neuron to NSPCs, the critical reasoning of using spike-triggered approaches in V2a-INs through pair recordings and evaluating the downstream impact to NSPCs is to determine effective communication between the two cell types, which our data do certainly suggest. Future studies may uncover a remaining question regarding the extent of the physiological relevance between the direct and indirect communication in modulating the NSPC activity after training. V2a-INs are regarded as fundamental components of the locomotor CPG and are therefore essential for initiating and maintaining locomotor rhythm^19–21,43,46,47^ and to provide the primary driving input to motoneurons during locomotion^43^. However, other local cholinergic interneurons, like the ones residing close to the central canal, exist in the spinal cord^45,69,70^, and we cannot rule out the possibility that their firing could produce value-related cholinergic input to NSPCs. Nevertheless, the data here link locomotor network activity^19–21^ to spinal cord neurogenesis and demonstrate an essential non-motor/non-neuronal function for the CPG.

## Methods

### Experimental animals

All animals were raised and kept in a core zebrafish facility at the Karolinska Institute following established practices. Adult zebrafish of both sexes (*Danio rerio*; *n* = 343 animals; 8–10 weeks old; length: 15–20 mm; weight: 0.04–0.06 g), wild type (AB/Tübingen), Tg(*Chx10:GFP*^*nns1*^), and Tg(*her4.1:GFP*) lines. Zebrafish of both sexes were used in all experiments. No selection criteria and blinding procedures were used to allocate zebrafish to any experimental group. The local Animal Research Ethical Committee (at Karolinska Institutet), Stockholm (Ethical permit no. 9248-2017) approved all experimental protocols, and were implemented under EU directive for the care and use of laboratory animals (2010/63/EU). All efforts were made to utilize only the minimum number of experimental animals necessary to obtain reliable scientific data.

### Training protocol

All animals used in the swim training paradigm had similar sizes (body length, BL; body depth, BD) and weights. Some of the designated animals (*n* = 8 zebrafish) were randomly selected and subjected to the critical speed (*U*_CRIT_) test, which measures the highest sustainable swimming speed a fish can reach using a commercially available swim tunnel (5 L; Loligo systems, SW10050). After determining the critical speed, animals were selected for the exercise training protocol, in which exercised/trained zebrafish (~25) swam at 60% of *U*_CRIT_ for 6 h per day, 5 days per week. To study the effect of training on animal growth, fish were trained for 6 consecutive weeks. At each time point (every 7 days), the fish were anesthetized in 0.03% tricaine methane sulfonate (MS-222, Sigma-Aldrich, E10521), and images of the body size were obtained. For all the other experiments, zebrafish were trained for 2 consecutive weeks. After the exercise period, fish were randomly assigned to a short-term experimental group (training) or long-term (rest) group. Animals of the recovery group were kept under standard conditions for 2 weeks. Afterward, all animals (training/rest) selected for anatomical investigations were anesthetized and processed for immunohistochemistry, as described in the “Immunohistochemistry” section. Trained animals for electrophysiological recordings were processed within the first three days after the end of the training.

### BrdU treatment

Animals were treated with 5-bromo-2ʹ-deoxyuridine (BrdU; Sigma-Aldrich, B5002) at a concentration of 0.7% in fish water for 2 h. BrdU is a nonradioactive analog of thymidine incorporated into proliferating cells’ DNA during the S phase of mitosis. Fish were then allowed to survive for another 22 h (short-term survival) or 2 weeks (long-term survival) before being processed for BrdU immunodetection. For the acute treatment, animals were treated with BrdU at a concentration of 0.7% in fish water for 1 h before analysis. In acute experiments described in Fig. 5a, animals were injected intraperitoneally (volume: 2 μl) with either saline, nicotine (300 μΜ; Sigma-Aldrich, SML1236), GABA (500 μΜ; Sigma-Aldrich, A2129), or gabazine (100 μΜ; Sigma-Aldrich, SR95531). Immediately after injection, the animals were treated with BrdU for 1 h, as described above.

### Descending neuron labeling

Zebrafish were anesthetized in 0.03% tricaine methane sulfonate (MS-222, Sigma-Aldrich, E10521). Retrograde labeling of descending spinal cord neurons located in spinal segments 1–3 was achieved through dye injections with biotinylated dextran (3000 MW; ThermoFisher, D7135) into segment 16 or 17. Animals were kept alive for at least 24 h after injection to allow retrograde transport of the tracer, deeply anesthetized with 0.1% MS-222, and the spinal cords were dissected and fixed in 4% paraformaldehyde (PFA) and 5% saturated picric acid (Sigma-Aldrich, P6744) in phosphate-buffered saline (PBS; 0.01 M, pH = 7.4; Santa Cruz Biotechnology, Inc., CAS30525-89-4) at 4 °C for 4–10 h. The tissue was then washed extensively with PBS and incubated in streptavidin conjugated to Alexa Fluor 488 (dilution 1:500, ThermoFisher, S32354), Alexa Fluor 555 (1:500, ThermoFisher, S32355), or Alexa Fluor 647 (dilution 1:500, ThermoFisher, S32357) overnight at 4 °C. Primary and secondary antibodies were applied as described in the “Immunohistochemistry” section. After thorough buffer rinses, the tissue was mounted on gelatin-coated microscope slides and cover-slipped with an anti-fade fluorescent mounting medium (Vectashield Hard Set, VectorLabs; H-1400).

### Pharmacology

For the experiments conducted to evaluate the impact of pharmacological agents on proliferation and neurogenesis in vivo, animals were anesthetized using 0.03% tricaine methane sulfonate (MS-222; Sigma-Aldrich, E10521) in fish water and injected intraperitoneally (volume: 2 μl) with saline, ACh (2 mM; Sigma-Aldrich, A6625), muscarine (50 μΜ; Sigma-Aldrich, M104), nicotine (300 μΜ; Sigma-Aldrich, SML1236), GABA (500 μΜ; Sigma-Aldrich, A2129), or gabazine (100 μΜ; Sigma-Aldrich, SR95531). Immediately after injection, the animals were treated with BrdU as described above (see “BrdU treatment” section). For ex vivo evaluation of NSPC receptor activation’s contribution to proliferation, animals were anesthetized and dissected as for the electrophysiological recordings. Isolated intact spinal cords were then transferred to a continuously aired chamber containing the pharmacological agents, and BrdU diluted in the extracellular solution used for electrophysiological recording. In some experiments the extracellular solution contained TTX (1 μM) to abolish the synaptic transmission in the spinal cord networks. After the pharmacological treatments, the animals and tissues were processed for immunodetection of the incorporated BrdU.

### Immunohistochemistry

All animals were deeply anesthetized with tricaine methane sulfonate (MS-222, Sigma-Aldrich, E10521). The spinal cords were then extracted and fixed in 4% paraformaldehyde (PFA) and 5% saturated picric acid (Sigma-Aldrich, P6744) in phosphate-buffered saline (PBS) (0.01 M; pH = 7.4, Santa Cruz Biotechnology, Inc., CAS30525-89-4) at 4 °C for 2–14 h. We performed immunolabeling in both whole-mount spinal cords and cryosections. For sections, the tissue was removed carefully and cryoprotected overnight in 30% (w/v) sucrose in PBS at 4 °C, embedded in Cryomount (Histolab, 45830) sectioning medium, rapidly frozen in dry-ice-cooled isopentane (2-methylbutane; Sigma-Aldrich, 277258) at approximately –35 °C, and stored at −80 °C until use. Transverse coronal plane cryosections (thickness: 20–25 μm) of the tissue were collected and processed for immunohistochemistry. For all sample types (whole-mount and cryosections), the tissue was washed 3 times for 5 min each in PBS. Nonspecific protein binding sites were blocked with 4% normal donkey serum (NDS; Sigma-Aldrich, D9663) with 1% bovine serum albumin (BSA; Sigma-Aldrich, A2153) and 1% Triton X-100 (Sigma-Aldrich, T8787) in PBS for 1 h at room temperature (RT). Primary antibodies (Supplementary Table 2) were diluted in 1% of the blocking solution and applied for 1–3 days at 4 °C. After thorough buffer rinses, the tissues were then incubated with the appropriate secondary antibodies (Supplementary Table 2) diluted 1:500 or with streptavidin conjugated to Alexa Fluor 488 (1:500, ThermoFisher, S32354), Alexa Fluor 555 (1:500, ThermoFisher, S32355), or Alexa Fluor 647 (1:500, ThermoFisher, S32357) in 1% Triton X-100 (Sigma-Aldrich, T8787) in PBS overnight at 4 °C. Finally, the tissue was thoroughly rinsed in PBS and cover-slipped with a hard fluorescent medium (VectorLabs; H-1400). To visualize the incorporated BrdU, DNA denaturation was performed by incubating the tissue in 2 N HCl for 30 min (sections) or 75 min (whole mounts) at 37 °C, followed by thorough washing in PBS. The standard immunodetection procedure described above was then applied.

### Electrophysiology

Adult zebrafish were cold-anesthetized in a slush of a frozen extracellular solution containing MS-222. The skin and muscles were removed to allow access to the spinal cord. The spinal cord was dissected out carefully and transferred to a recording chamber that was continuously perfused with an extracellular solution containing 135.2 mM NaCl, 2.9 mM KCl, 2.1 mM CaCl2, 10 mM HEPES, and 10 mM glucose at pH 7.8 (adjusted with NaOH) and an osmolarity of 290 mOsm. For whole-cell intracellular recordings of NSPCs in voltage-clamp mode, electrodes (resistance, 3–5 MΩ) were pulled from borosilicate glass (outer diameter, 1.5 mm; inner diameter, 0.87 mm; Hilgenberg) on a micropipette puller (model P-97, Sutter Instruments) and filled with an intracellular solution containing 120 mM K-gluconate, 5 mM KCl, 10 mM HEPES, 4 mM Mg2ATP, 0.3 mM Na4GTP, and 10 mM Na-phosphocreatine at pH 7.4 (adjusted with KOH) and an osmolarity of 275 mOsm. Cells were visualized using a microscope (LNscope; Luigs & Neumann) equipped with a CCD camera (Lumenera) and explicitly targeted. Intracellular patch-clamp electrodes were advanced to the stem/progenitor cells using a motorized micromanipulator (Luigs & Neumann) while applying constant positive pressure. Intracellular signals were amplified with a MultiClamp 700B intracellular amplifier (Molecular Devices). All cells were clamped at –70 mV throughout all voltage-clamp recordings. All experiments were performed at RT (23 °C). The following drugs (prepared by diluting stock solutions in distilled water) were added (singly or in combinations mentioned in the text) to the physiological solution: acetylcholine (ACh, 100 μM or 5 mM; Sigma-Aldrich, A6625), GABA (γ-aminobutyric acid, 1 or 15 mM; Sigma-Aldrich, A2129), gabazine (10 μM; Sigma-Aldrich, SR95531), glutamate (5 mM; Sigma-Aldrich), glycine (1 mM; Sigma-Aldrich, G2879), methyllycaconitine (MLA, 10 μΜ; Sigma-Aldrich, M168), muscarine (500 μΜ; Sigma-Aldrich, M104), nicotine (100 μM; Sigma-Aldrich, N3876 and SML1236), *N*-methyl-*D*-aspartate (NMDA, 100 μM; Sigma-Aldrich, M3262), serotonin (1 mM; Sigma-Aldrich, H9523), and tetrodotoxin (TTX, 1 μΜ; Sigma-Aldrich, T8024). For the evaluation of the activity of the NSPCs during fictive locomotion (Fig. 2a), we used the adult zebrafish ex vivo preparation^42,71^. Extracellular recordings were performed from the motor nerves. Activation of the locomotion was induced by extracellular stimulation (using a train of 10 pulses: 1 Hz) applied via a glass pipette placed at the junction between the brain and the spinal cord. Recordings were made from both ipsilateral and contralateral located NSPCs and motor nerves (Fig. 2b). The local spinal neuron activation was triggered by extracellular stimulation (using a train of 10 pulses: 20 Hz) applied via a glass pipette (Fig. 3c). Activation of the long descending spinal neurons was induced by extracellular stimulation (using a train of 10 pulses: 20 Hz) applied via a glass pipette placed 5–6 segments rostral to whole-cell NSPC recording the adult zebrafish spinal cord (Fig. 4i). To attenuate and potentially block the polysynaptic transmission, we used 2.5x HiDi (a high concentration of divalent cations) solution or mephenesin (1 mM; Sigma-Aldrich, 286567) a possible polysynaptic blocker applied for at least 20 min before all the recordings. For dual whole-cell recordings of V2a-INs and progenitors/stem cells, two patch-clamp electrodes were advanced from opposite directions into the spinal cord to record cells separated by at least five segments (inter-segmental recordings) or from the same spinal segment (intra-segmental recordings). Single and multiple short-duration (0.5 ms) suprathreshold and subthreshold current pulses were used to stimulate presynaptic V2a interneurons and record responses in stem/progenitor cells. All dual whole-cell recordings are presented as averages of 20–35 sweeps. Only NSPCs (GFP^+^) that had stable resting membrane potentials at or below −60 mV did not fire action potentials upon strong depolarizations (>0 mV) and showed minimal changes in resistance (<5%) were included in this study. In all recordings, the EPSC events were detected and analyzed in a semi-automatic (supervised) fashion after baseline subtraction using AxoGraph (version X 1.5.4; AxoGraph Scientific, Sydney, Australia; RRID: SCR_014284) or Clampfit (version 10.6; Molecular Devices). The EPSC amplitude was calculated as the difference between the baseline and the peak of the event.

### Spinal cord injury

Adult zebrafish were anesthetized in 0.03% tricaine methane sulfonate (MS- 222; Sigma-Aldrich) before subjection to the spinal cord injury, which involved complete transection of the spinal cord segment 15 with a micro knife (10318-14; Fine Science Tools) under constant visual control. Once the lesion was completed, the spinally transected animals were injected intraperitoneally (volume: 2 μl) with BrdU solution (Sigma; 0.2 mg/g body weight) containing either saline (control), nicotine (300 μΜ; Sigma-Aldrich, SML1236), or gabazine (100 μΜ; Sigma-Aldrich, SR95531). All animals were kept in freshwater under standard conditions and received two additional intraperitoneal injections of the drugs every 4 days, as described in Fig. 6a.

### Critical speed test

All post-injured animals (12 days after injury) were subjected to the critical speed (*U*_CRIT_) test using a commercially available swim tunnel (5 L; Loligo systems, SW10050). Critical speed (*U*_CRIT_) is a measure of the highest sustainable swimming speed that a fish can reach. The zebrafish were subjected to time intervals (2 min) of increased water flow velocity (increments of 4.5 cm/s) until the fish could not swim against the water current^31^. The critical speed was normalized to the experimental animals’ body length (BL) and is given as BL/s.

### Analysis

Morphometric analysis of the adult zebrafish was performed in images acquired with an HD camera (MC120, Leica) attached to a stereomicroscope (M60, Leica). The body size (total length, TL) of each animal was quantified using ImageJ. All immunodetections of whole-mount images of the adult zebrafish spinal cord preparations were acquired using an LSM 800 laser scanning confocal microscope (Zeiss) with a 40x objective (oil immersion). Each examined whole-mount spinal cord hemisegment was scanned from the ipsilateral side to the contralateral side at the contralateral primary motoneurons level to ensure the central canal region’s acquisition, generating a z-stack (z-step size = 0.3–0.5 μm). All neurons, including *her4*:GFP^+^ stem/progenitor cells and newborn cells, were counted in spinal hemisegment 15 or 16. The neurons’ somata and cells’ relative positions (*XYZ* coordinates) within the spinal cord were determined (using the lateral, dorsal, and ventral edges of the cord as landmarks) using ImageJ (cell counter plugin). Soma sizes and numbers were also measured using ImageJ. All whole-mount data are presented as the number of cells or neurons in each analyzed hemisegment. The central canal region was defined as the area in the midline between the contralateral primary motoneurons. Analysis and quantifications of the BrdU^+^ cell differentiation profiles were performed using 6 coronal sections (20 μm thick, 20 μm intervals) of the spinal cord segments 14–16. All quantifications in the injured animals were performed in an area of 150 μm length, located 50–60 μm rostrally from the injured site. The probability matrix of the V2a-IN processes in the central canal area was generated from multiple data (~7 sections/animal; *n* = 15 zebrafish) using Origin 8 (OriginLab, Northampton, MA, USA). To enhance visualization of our data, most of the whole-mount images presented here were prepared by merging subsets of the original z-stacks. Most single channel images showing the BrdU^+^ were inverted to allow better visualization. Colocalizations were detected by visual identification of structures whose color reflects the combined contribution of two or more antibodies in the merged image. Most of the presented traces were low-pass filtered (Gaussian, 11-21 coefficients) using Clampfit (version 11.0; Molecular Devices). All figures and graphs were prepared with Adobe Photoshop and Adobe Illustrator (Adobe Systems Inc., San Jose, CA, USA). Digital modifications of the images (brightness and contrast) were minimal to diminish the potential distortion of biological information. All double-labeled immunofluorescence images were converted to magenta-green to improve visualization of the results for color-blind readers.

### Statistics and reproducibility

The significance of differences between the means in experimental groups and conditions was analyzed using parametric tests such as the two-tailed unpaired or paired Student’s *t*-test and one-way ANOVA (ordinary) followed by post hoc Tukey’s test or Dunnett’s multiple comparison test, using Prism (GraphPad Software Inc.). Significance levels indicated in all figures are as follows: ^*^P < 0.05, ^**^P < 0.01, ^***^P < 0.001, ^****^P < 0.0001. All data are presented as mean ± s.e.m. (standard error of mean) or as box plots showing the median, 25th, and 75th percentile (box and line) and minimal and maximal values (whiskers). Finally, the *n* values indicate the final number of validated animals per group, cells, or events that were evaluated and presented in detail in Supplementary Table 1. All experiments were carried out independently 2–5 times from different investigators.

### Reporting summary

Further information on research design is available in the Nature Research Reporting Summary linked to this article.

## Supplementary information

Supplementary information.

Reporting summary.

## Data Availability

All data used for the analyses presented in this study are included in Supplementary Table 1. Source data are provided with this paper.

## Source data

Source data file.
