# Supplementary material for: Locomotion dependent neuron-glia interactions control neurogenesis and regeneration in the adult zebrafish spinal cord
Source: Nat Commun. 2021 Aug 11;12:4857. doi: 10.1038/s41467-021-25052-1 (PMC8357999; doi:10.1038/s41467-021-25052-1)
Supplement: Supplementary file 1 — Supplementary information. [file 41467_2021_25052_MOESM1_ESM.pdf]

**Supplementary Information:**

# Locomotion dependent neuron-glia interactions control neurogenesis and regeneration in the adult zebrafish spinal cord

Weipang Chang, Andrea Pedroni, Maria Bertuzzi, Caghan Kizil, András Simon, Konstantinos Ampatzis

Supplementary Figures: 1-9
Supplementary Tables: 1-2

**Supplementary figures:**

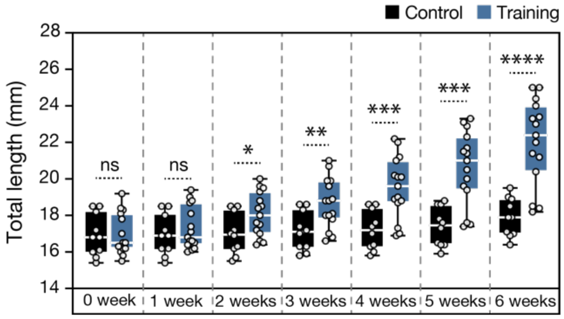


**Supplementary Fig. 1. Physical activity increases animal growth.** Quantification of the animal total length (TL) between the untrained (control; n=10) and trained (n=15) adult zebrafish. Data are presented as box plots showing the median with 25/75 percentile (box and line) and minimum–maximum (whiskers). *$P<0.05$; **$P<0.01$; ***$P<0.001$; ****$P<0.0001$; ns, not significant. For detailed statistics, see supplementary Table 1.

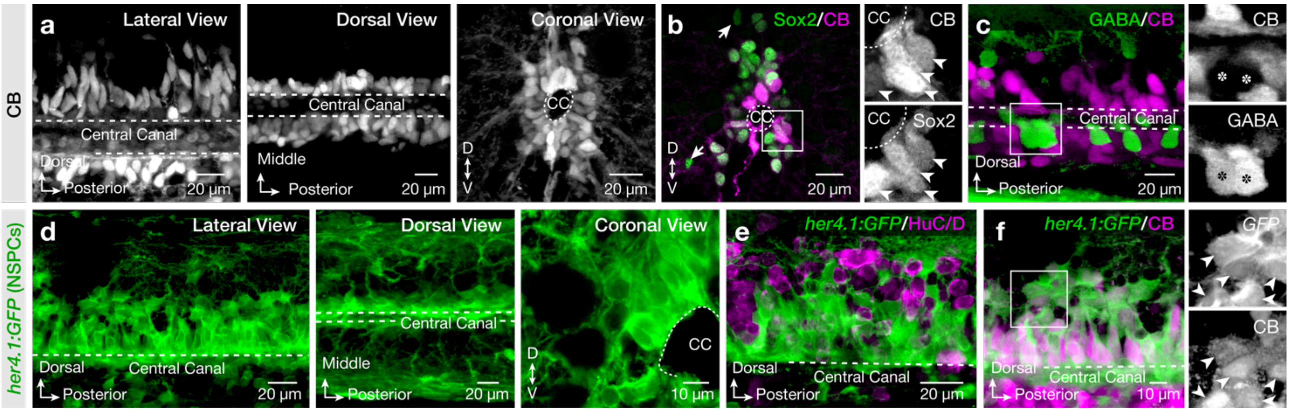


**Supplementary Fig. 2. Identification of spinal cord neural stem/progenitor cells (NSPCs).** **a** Distribution pattern of the calbindin$^{+}$ (CB$^{+}$) immunolabeled cells in the central canal (cc) area. **b** High colocalization of CB (magenta) with the stem cell marker Sox2 (green). Arrows indicate the Sox2$^{+}$CB$^{-}$ cells. Arrowheads in the inset indicate the double-labeled cells. **c** The cerebrospinal fluid contacting neurons (GABA$^{+}$, green) do not express CB (magenta). Asterisks indicate the GABA$^{+}$CB$^{-}$ neurons. **d** Expression pattern of *GFP* (green) driven by the radial glial promoter *her4.1* in the adult zebrafish spinal cord defining the spinal NSPCs. **e** GFP$^{+}$ NSPCs (green) do not express the neuronal marker HuC/D (magenta). **f** All *her4.1*$^{+}$ cells (NSPCs, green) are CB$^{+}$ (magenta). Arrowheads in the inset indicate the double-labeled cells. CB, calbindin D-28K; CC, central canal; D, dorsal; GABA, γ-aminobutyric acid; GFP, green fluorescent protein; her4.1, hairy-related 4, tandem duplicate 1; HuC/D, elav3+4; NSPC, neural stem/progenitor cell; Sox2, sex-determining region Y-box 2; V, ventral.

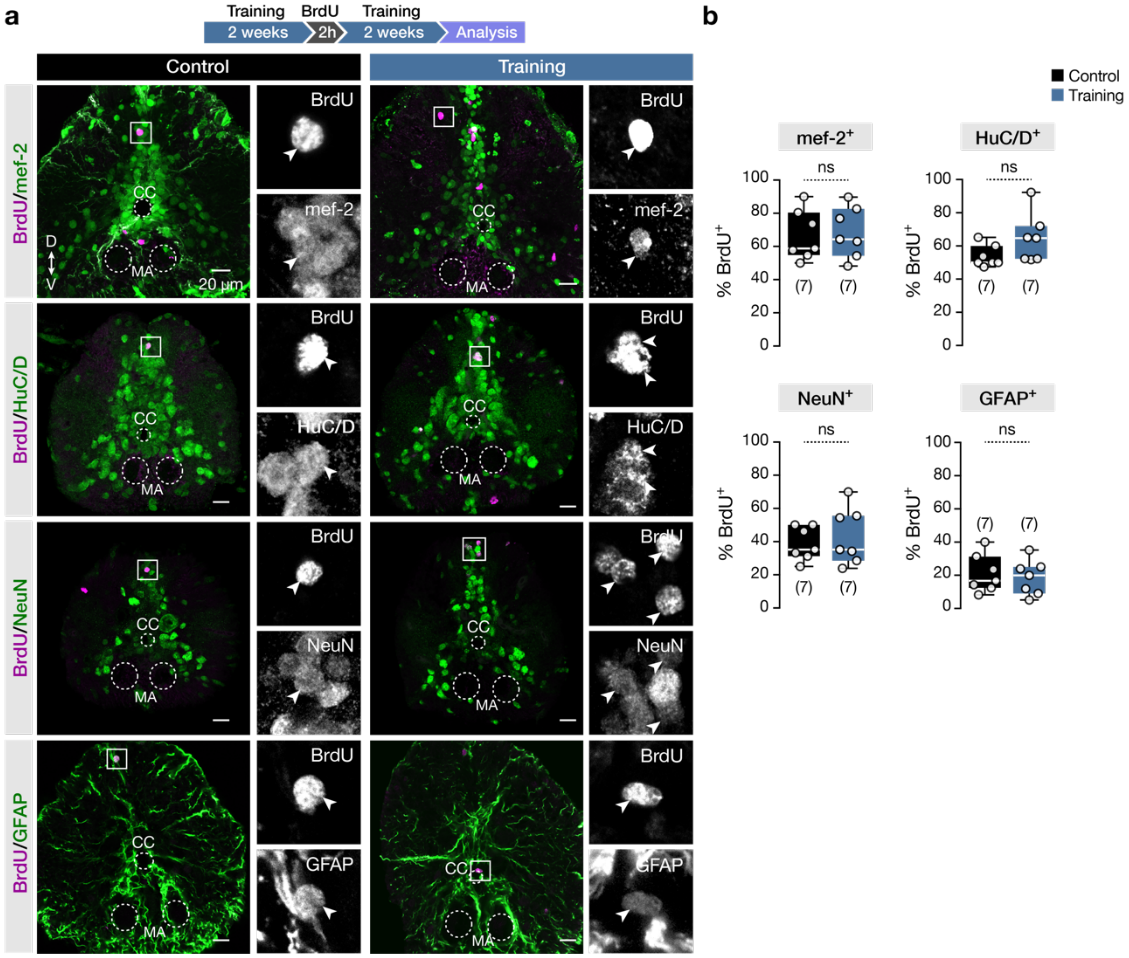


**Supplementary Fig. 3. Differentiation profile of BrdU⁺ cells. a** Coronal sections of the adult zebrafish spinal cord used for immunodetection of BrdU⁺ cells (magenta), expressing neuronal (mef-2, HuC/D, NeuN; green) or GFAP (green) markers in control and trained animals. Arrowheads indicate double-labeled cells. **b** Quantification of the proportion of the BrdU⁺ cells that are also mef-2⁺, HuC/D⁺, NeuN⁺, or GFAP⁺ in control and trained animals. A majority of BrdU⁺ cells express neuronal markers indication neuronal differentiation. BrdU, 5-Bromo-2'-Deoxyuridine; CC, central canal; D, dorsal; MA, GFAP, Glial fibrillary acidic protein; Mauthner axon; mef-2, myocyte enhancer factor-2; NeuN, neuronal nuclei; V, ventral. Data are presented as box plots showing the median with 25/75 percentile (box and line) and minimum–maximum (whiskers). ns, not significant. For detailed statistics, see supplementary Table 1.

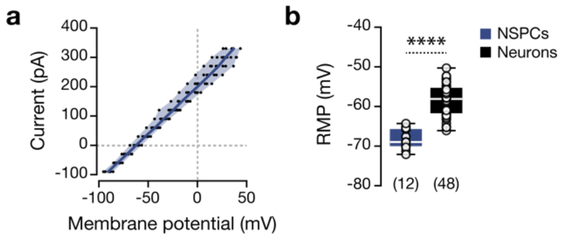


**Supplementary Fig. 4. Cellular properties of spinal NSPCs. a** Current-Voltage (I-V) relationships for the NSPCs (*her4.1:GFP*$^+$) obtained from current-clamp recordings. **b** Resting membrane potential differences between the NSPCs (*her4.1:GFP*$^+$) and spinal cord neurons (V2a-INs and motoneurons; *n*: number of recorded cells; *t*-test: t = 8.226, df = 58, *P*=2.563E-11). NSPC, neural stem/progenitor cell; RMP, resting membrane potential. Data are presented as box plots showing the median with 25/75 percentile (box and line) and minimum–maximum (whiskers). ****$P$<0.0001. For detailed statistics, see supplementary Table 1.

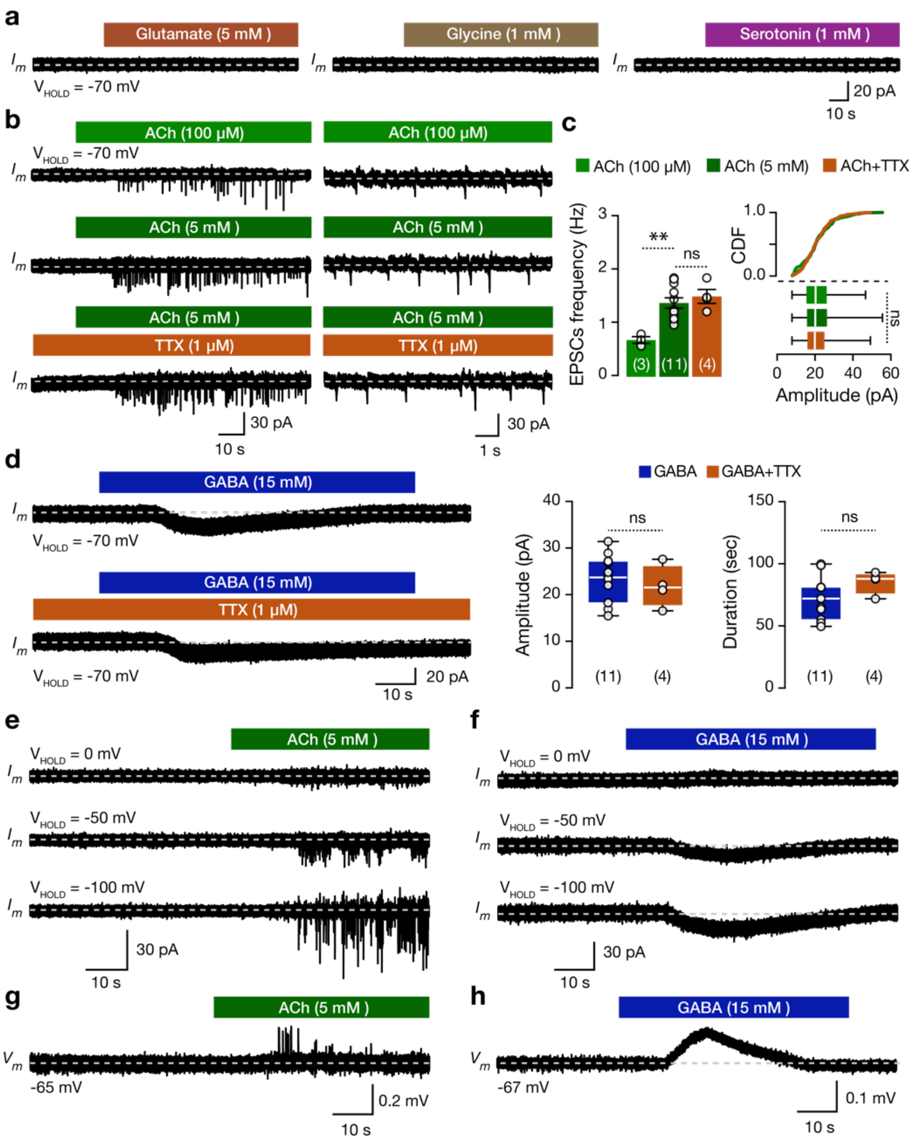


**Supplementary Fig. 5. Cholinergic and GABAergic responses by NSPCs in the adult spinal cord. a** Voltage-clamp recordings followed by bath application of glutamate (0 out of 8), glycine (0 out of 6), and serotonin (0 out of 7) do not evoke any apparent response in NSPCs. **b** Representative traces show the induced currents in NSPCs after bath application of ACh (100μM or 5mM) and in the presence of TTX (1 μM). (**c**) ACh treatment induced a dose-dependent increase in EPSC frequency, but the amplitudes of the EPSCs were independent of the ACh concentration or the presence of TTX (*n*: number of recorded NSPCs). **d** Sample traces show the induced tonic activity currents in NSPCs after bath application of GABA (5mM) in presence of TTX (1 μM). Quantification of the response amplitude and duration in control and TTX (*n*: number of recorded NSPCs). **e-f** Sample traces showing the differences between the ACh and GABA induced responses in the spinal cord NSPCs at different holding potentials. **g-h** Current-clamp NSPCs recordings show that ACh application generated many EPSPs, whereas GABA induced a tonic membrane depolarization. ACh, acetylcholine; CDF, cumulative distribution frequencies; GABA, γ-aminobutyric acid; TTX, tetrodotoxin. The dashed gray line represents the baseline. Data are presented as means ± s.e.m. and as box plots showing the median with 25/75 percentile (box and line) and minimum-maximum (whiskers). **$P$<0.01; ns, not significant. For detailed statistics, see supplementary Table 1.

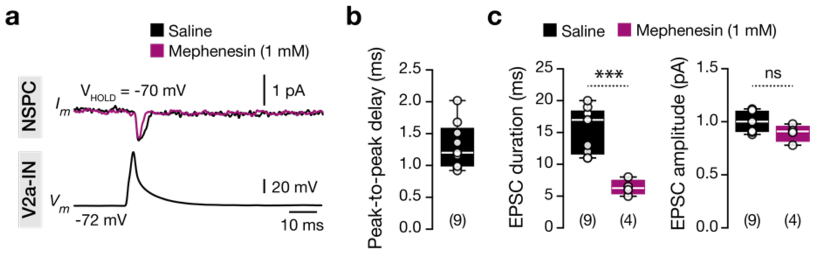


**Supplementary Fig. 6. Analysis of the inter-segmental connectivity between the V2a-INs and the NSPCs. a** Example traces (lowpass filtered) showing the changes in the evoked EPSCs in a postsynaptic NSPC in control (saline, black trace) and after application of the potential polysynaptic blocker mephenesin (magenta). **b** Analysis of the synaptic delay between the V2a-INs and the NSPCs postsynaptic responses suggesting that are likely monosynaptic (*n*: number of recorded NSPC/V2a-IN pairs). **c** Quantification of EPSCs duration and amplitude before and after the application of mephenesin suggesting a mixture of monosynaptic (direct) and polysynaptic (indirect) connections (*n*: number of recorded NSPC/V2a-IN pairs). NSPC, neural stem/progenitor cell. Data are presented as box plots showing the median with 25/75 percentile (box and line) and minimum-maximum (whiskers). ***$P$<0.001; ns, not significant. For detailed statistics, see supplementary Table 1.

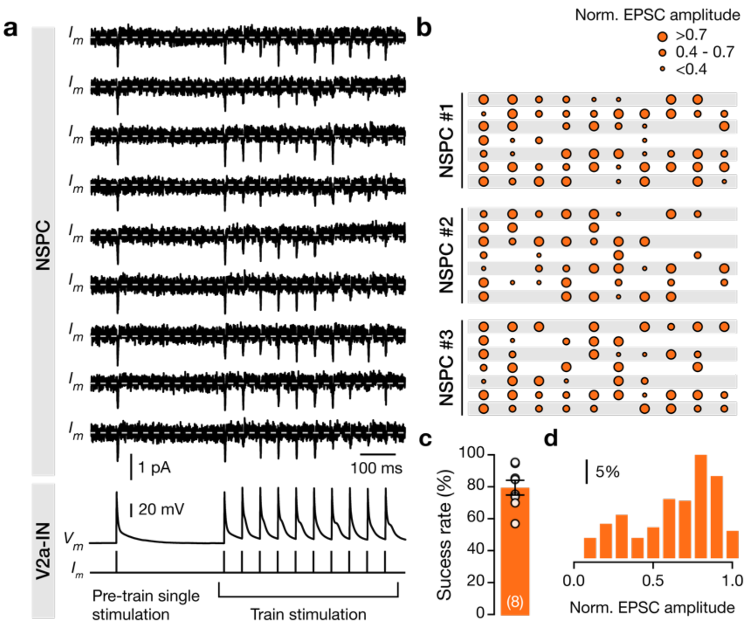


**Supplementary Fig. 7. Synaptic connection reliability between V2a-INs and the NSPCs. a** Example traces of individual sweeps ($n$=9) showing the failures and changes in the evoked EPSCs in postsynaptic stem/progenitor cells during the stimulation train. **b** Analysis from three V2a-IN / NSPC pairs showing the failures and the normalized EPSC amplitude changes during the train of action potentials evoked in V2a-INs. **c-d** Success rate analysis (%) and frequency distribution of the evoked EPSC amplitudes during the train of action potential evoked in V2a-INs ($n$: number of recorded NSPC/V2a-IN pairs). In all cases, the EPSC amplitudes were normalized to the EPSC amplitude of the pre-train single stimulation. The dashed gray line represents the baseline. NSPC, neural stem/progenitor cell. Data are presented as means ± s.e.m. For detailed statistics, see supplementary Table 1.

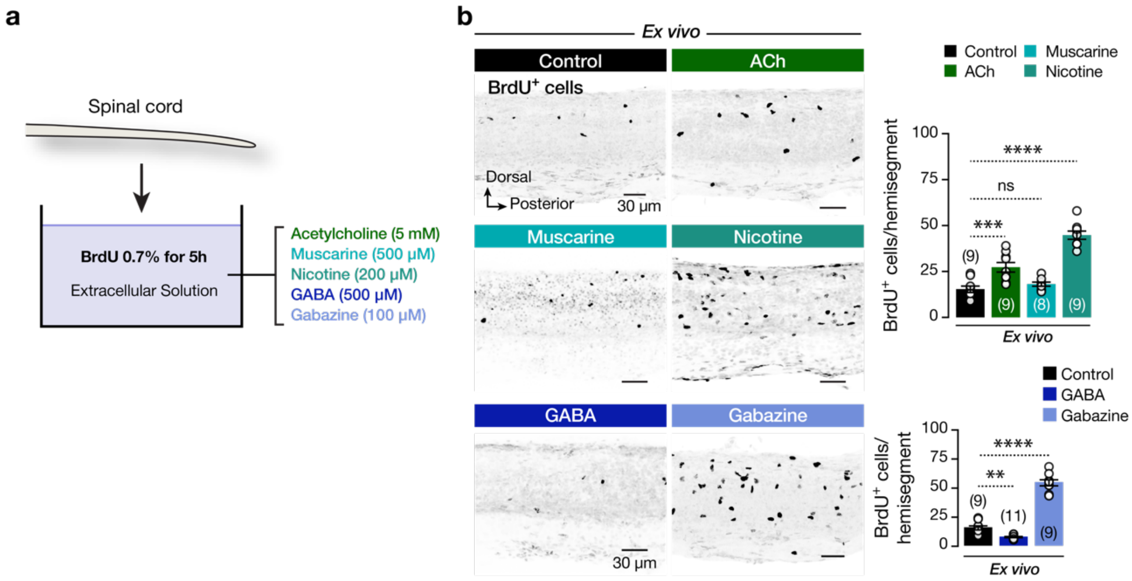


**Supplementary Fig. 8. *Ex vivo* cholinergic and GABAergic modulation of proliferation in the spinal cord. a** Experimental setup for the *ex vivo* investigation of proliferation in intact isolated spinal cords. **b** Whole-mount confocal microphotographs illustrating BrdU-incorporation per hemisegment following pharmacological treatments and quantifications. ACh, muscarine, and nicotine increased the number of BrdU$^+$ cells. Administration of GABA or gabazine indicated a role for GABA$_A$ receptors in maintaining NSPC quiescence. ACh, acetylcholine; BrdU, 5-Bromo-2'-Deoxyuridine; GABA, γ-aminobutyric acid. Data are presented as means ± s.e.m. ***P*<0.01; ****P*<0.001; *****P*<0.0001; ns, not significant. For detailed statistics, see supplementary Table 1.

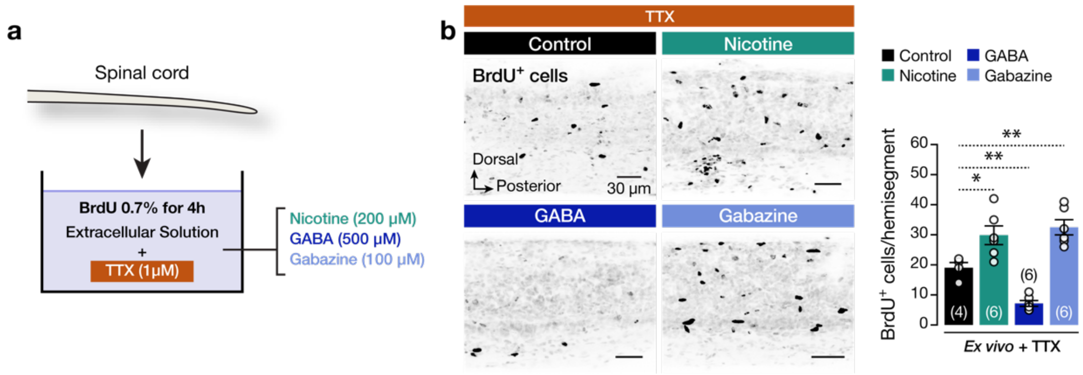


**Supplementary Fig. 9. *Ex vivo* cholinergic and GABAergic modulation of spinal cord proliferation in spike eliminating environment. a** Experimental setup for the *ex vivo* investigation of proliferation in intact isolated spinal cords in a presence of tetrodotoxin. **b** Whole-mount confocal microphotographs illustrating the detected BrdU$^+$ per hemisegment following pharmacological treatments and followed by quantifications. BrdU, 5-Bromo-2'-Deoxyuridine; GABA, γ-aminobutyric acid; TTX, tetrodotoxin. Data are presented as means ± s.e.m. **P*<0.05; ***P*<0.01. For detailed statistics, see supplementary Table 1.

**Supplementary Table 1. Detailed statistics**

| Figure | Statistics | Result | Post-hoc Test | comparison | Significance | *P-value* |
|---|---|---|---|---|---|---|
| **1c** | One-way ANOVA | $F_{(2, 24)} = 60.23$, $P < 0.0001$ | Dunnett's test | Control<br>Training | **** | $P_{adj} < 0.0001$ |
| | | | | Control<br>Rest | ns | $P_{adj} = 0.206$ |
| | Descriptive | Control (*n* = 10 zebrafish): 14.5 ± 1.376<br>Training (*n* = 9 zebrafish): 42.44 ± 3.532<br>Rest (*n* = 8 zebrafish): 9.25 ± 0.959 | | | | |
| **1f** | Unpaired *t*-test | t = 3.946, df = 10 (Two-tailed) | | Control<br>Training | ** | $P = 0.0027$ |
| | Descriptive | Control (*n* = 5 zebrafish): 1.66 ± 0.235<br>Training (*n* = 7 zebrafish): 4.09 ± 0.486 | | | | |
| **1g**<br>mef-2 | Unpaired *t*-test | t = 6.495, df = 12 (Two-tailed) | | Control<br>Training | **** | $P < 0.0001$ |
| | Descriptive | Control (*n* = 7 zebrafish): 0.952 ± 0.086<br>Training (*n* = 7 zebrafish): 2.69 ± 0.253 | | | | |
| **1g**<br>HuC/D | Unpaired *t*-test | t = 5.536, df = 12 (Two-tailed) | | Control<br>Training | *** | $P = 0.0001$ |
| | Descriptive | Control (*n* = 7 zebrafish): 0.857 ± 0.111<br>Training (*n* = 7 zebrafish): 2.19 ± 0.213 | | | | |
| **1g**<br>NeuN | Unpaired *t*-test | t = 4.475, df = 12 (Two-tailed) | | Control<br>Training | *** | $P = 0.0008$ |
| | Descriptive | Control (*n* = 7 zebrafish): 0.595 ± 0.071<br>Training (*n* = 7 zebrafish): 1.619 ± 0.217 | | | | |
| **2d** | Linear regression | $R^2 = 0.2124$<br>Sy.x = 1.296<br>Equation: Y= -0.6834*X + 7.865 | | | | |
| **2e** | Linear regression | $R^2 = 0.9098$<br>Sy.x = 0.4203<br>Equation: Y=0.3552*X – 1.09 | | | | |
| **3a**<br>Frequency | One-way ANOVA | $F_{(2, 22)} = 17.15$, $P < 0.0001$ | Tukey's test | ACh<br>Muscarine | **** | $P_{adj} < 0.0001$ |
| | | | | ACh<br>Nicotine | ns | $P_{adj} = 0.9978$ |
| | | | | Muscarine<br>Nicotine | *** | $P_{adj} = 0.0001$ |
| | Descriptive | ACh (*n* = 11 cells): 1.362 ± 0.098<br>Muscarine (*n* = 6 cells): 0.694 ± 0.045<br>Nicotine (*n* = 8 cells): 1.355 ± 0.054 | | | | |
| **3a**<br>Amplitude | One-way ANOVA | $F_{(2, 573)} = 66.56$, $P < 0.0001$ | Tukey's test | ACh<br>Muscarine | **** | $P_{adj} < 0.0001$ |
| | | | | ACh<br>Nicotine | ns | $P_{adj} = 0.4183$ |
| | | | | Muscarine<br>Nicotine | **** | $P_{adj} < 0.0001$ |
| | Descriptive | ACh (*n* = 294 EPSCs): 21.48 ± 0.46<br>Muscarine (*n* = 88 EPSCs): 11.85 ± 0.295<br>Nicotine (*n* = 194 EPSCs): 20.67 ± 0.494 | | | | |
| **3b**<br>Amplitude | One-way ANOVA | $F_{(2, 18)} = 46.38$, $P < 0.0001$ | Tukey's test | GABA<br>Muscimol | ns | $P_{adj} = 0.616$ |
| | | | | GABA<br>GABA + Gabazine | **** | $P_{adj} < 0.0001$ |
| | | | | Muscimol<br>GABA + Gabazine | **** | $P_{adj} < 0.0001$ |
| | Descriptive | GABA (*n* = 11 cells): 23.55 ± 1.524<br>Muscimol (*n* = 6 cells): 25.75 ± 2.0<br>GABA + Gabazine (*n* = 4 cells): 0.0 ± 0.0 | | | | |
| **3b**<br>Duration | One-way ANOVA | $F_{(2, 18)} = 44.16$, $P < 0.0001$ | Tukey's test | GABA<br>Muscimol | ns | $P_{adj} = 0.9451$ |

| | | | | | | |
|---|---|---|---|---|---|---|
| | | | | GABA<br>GABA + Gabazine | **** | $P_{adj}$ < 0.0001 |
| | | | | Muscimol<br>GABA + Gabazine | **** | $P_{adj}$ < 0.0001 |
| | Descriptive | GABA (*n* = 11 cells): 72.73 ± 5.233<br>Muscimol (*n* = 6 cells): 75.02 ± 4.319<br>GABA + Gabazine (*n* = 4 cells): 0.0 ± 0.0 | | | | |
| **3c**<br>µ EPSCs number | Paired<br>*t*-test | t = 2.584, df = 4 (Two-tailed) | | Control<br>Mephenesin | ns | *P* = 0.0610 |
| | Descriptive | Control (*n* = 5 cells): 0.804 ± 0.057<br>Mephenesin (*n* = 5 cells): 0.678 ± 0.042 | | | | |
| **3c**<br>µ EPSCs amplitude | Paired<br>*t*-test | t = 0.191, df = 4 (Two-tailed) | | Control<br>Mephenesin | ns | *P* = 0.8575 |
| | Descriptive | Control (*n* = 5 cells): 8.608 ± 0.985<br>Mephenesin (*n* = 5 cells): 8.553 ± 0.964 | | | | |
| **4b** | Descriptive | (*n* = 8 zebrafish): 41.81 ± 3.62 | | | | |
| **4d** | Descriptive | (*n* = 8 zebrafish)<br>V2a-INs: 25 out of 25 neurons (100%)<br>Non V2a-INs: 0 out of 25 neurons (0%) | | | | |
| **4e**<br>Number of neurons | Descriptive | (*n* = 8 zebrafish)<br>Number of neurons/hemisegment: 3.125 ± 0.226 | | | | |
| **4e**<br>Soma size | Descriptive | (*n* = 25 neurons from 8 zebrafish)<br>78.99 ± 3.189 | | | | |
| **4f**<br>Connectivity | Descriptive | Intra-segmental: 0 out of 15 (0.0%)<br>Inter-segmental: 11 out of 50 (22%) | | | | |
| **4i**<br>responses | Descriptive | Control (*n* = 9 cells)<br>Responding: 3 (37.5%)<br>Not Responding: 5 (62.5%)<br>Training (*n* = 12 cells)<br>Responding: 7 (58.33%)<br>Not Responding: 5 (41.67%) | | | | |
| **4i**<br>EPSCs | Unpaired<br>*t*-test | t = 9.524, df = 210 (Two-tailed) | | Control<br>Training | **** | *P* < 0.0001 |
| | Descriptive | Control (*n* = 87): 0.942 ± 0.082<br>Training (*n* = 125): 2.376 ± 0.111 | | | | |
| **5a** | One-way ANOVA | $F_{(3, 215)}$ = 59.88, *P* < 0.0001 | Tukey's test | Saline<br>Nicotine | **** | $P_{adj}$ < 0.0001 |
| | | | | Saline<br>GABA | * | $P_{adj}$ = 0.013 |
| | | | | Saline<br>Gabazine | *** | $P_{adj}$ = 0.0004 |
| | | | | Nicotine<br>GABA | **** | $P_{adj}$ < 0.0001 |
| | | | | Nicotine<br>Gabazine | ns | $P_{adj}$ = 0.265 |
| | | | | GABA<br>Gabazine | **** | $P_{adj}$ < 0.0001 |
| | Descriptive | Saline (*n* = 4 zebrafish): 1.917 ± 0.515<br>Nicotine (*n* = 5 zebrafish): 5.267 ± 0.286<br>GABA (*n* = 6 zebrafish): 0.383 ± 0.183<br>Gabazine (*n* = 4 zebrafish): 4.417 ± 0.25 | | | | |
| **5b**<br>Cholinergic | One-way ANOVA | $F_{(3, 32)}$ = 128.4, *P* < 0.0001 | Dunnett's test | Saline<br>ACh | **** | $P_{adj}$ < 0.0001 |
| | | | | Saline<br>Muscarine | ** | $P_{adj}$ = 0.0023 |
| | | | | Saline<br>Nicotine | **** | $P_{adj}$ < 0.0001 |
| | Descriptive | Saline (*n* = 13 zebrafish): 17.85 ± 1.372<br>ACh (*n* = 9 zebrafish): 34.44 ± 2.873<br>Muscarine (*n* = 8 zebrafish): 29 ± 1.464 | | | | |

| | | | | | | |
|---|---|---|---|---|---|---|
| | | Nicotine (*n* = 6 zebrafish): 81.67 ± 3.739 | | | | |
| **5b** GABAergic | One-way ANOVA | $F_{(2, 21)}$ = 270.2, *P* < 0.0001 | Dunnett's test | Saline GABA | *** | $P_{adj}$ = 0.0002 |
| | | | | Saline Gabazine | **** | $P_{adj}$ < 0.0001 |
| | Descriptive | Saline (*n* = 13 zebrafish): 17.85 ± 1.372<br>GABA (*n* = 6 zebrafish): 3.66 ± 1.909<br>Gabazine (*n* = 5 zebrafish): 81.6 ± 4.155 | | | | |
| **5c** | One-way ANOVA | $F_{(4, 31)}$ = 141.3, *P* < 0.0001 | Tukey's test | Saline Nicotine+Gabazine | **** | $P_{adj}$ < 0.0001 |
| | | | | Saline Nicotine | **** | $P_{adj}$ < 0.0001 |
| | | | | Saline Gabazine | **** | $P_{adj}$ < 0.0001 |
| | | | | Saline Nicotine+GABA | ns | $P_{adj}$ = 0.4206 |
| | | | | Nicotine Gabazine | ns | $P_{adj}$ > 0.9999 |
| | | | | Nicotine Nicotine+Gabazine | ns | $P_{adj}$ = 0.9814 |
| | | | | Nicotine Nicotine+GABA | **** | $P_{adj}$ < 0.0001 |
| | | | | Gabazine Nicotine+Gabazine | ns | $P_{adj}$ = 0.9828 |
| | | | | Gabazine Nicotine+GABA | **** | $P_{adj}$ < 0.0001 |
| | | | | Nicotine+Gabazine Nicotine+GABA | **** | $P_{adj}$ < 0.0001 |
| | Descriptive | Saline (*n* = 13 zebrafish): 17.85 ± 1.372<br>Nicotine+gabazine (*n* = 6 zebrafish): 84.17 ± 3.28<br>Nicotine (*n* = 6 zebrafish): 81.67 ± 3.739<br>Gabazine (*n* = 5 zebrafish): 81.6 ± 4.155<br>Nicotine+GABA (*n* = 6 zebrafish): 24.67 ± 4.302 | | | | |
| **5d** Frequency | Unpaired *t*-test | t = 0.418, df = 16 (Two-tailed) | | Control Training | ns | *P* = 0.6809 |
| | Descriptive | Control (*n* = 11 cells): 1.362 ± 0.098<br>Training (*n* = 7 cells): 1.429 ± 0.1253 | | | | |
| **5d** Amplitude | Unpaired *t*-test | t = 0.379, df = 543 (Two-tailed) | | Control Training | ns | *P* = 0.3791 |
| | Descriptive | Control (*n* = 325 EPSCs): 20.97 ± 0.436<br>Training (*n* = 220 EPSCs): 20.39 ± 0.48 | | | | |
| **5e** Amplitude | Unpaired *t*-test | t = 4.295, df = 15 (Two-tailed) | | Control Training | *** | *P* = 0.0006 |
| | Descriptive | Control (*n* = 11 cells): 23.55 ± 1.524<br>Training (*n* = 6 cells): 13.1 ± 1.723 | | | | |
| **5e** Duration | Unpaired *t*-test | t = 1.351, df = 15 (Two-tailed) | | Control Training | ns | *P* = 0.1967 |
| | Descriptive | Control (*n* = 11 cells): 72.73 ± 5.233<br>Training (*n* = 6 cells): 58.75 ± 10.36 | | | | |
| **6d** | One-way ANOVA | $F_{(2, 16)}$ = 7.954, *P* = 0.004 | Dunnett's test | Saline Nicotine | ** | $P_{adj}$ = 0.0026 |
| | | | | Saline Gabazine | * | $P_{adj}$ = 0.0336 |
| | Descriptive | Saline (*n* = 7 zebrafish): 56.86 ± 2.521<br>Nicotine (*n* = 6 zebrafish): 74.0 ± 4.091<br>Gabazine (*n* = 6 zebrafish): 68.5 ± 2.849 | | | | |
| **6e** | One-way ANOVA | $F_{(2, 16)}$ = 5.741, *P* = 0.0132 | Dunnett's test | Saline Nicotine | * | $P_{adj}$ = 0.0127 |
| | | | | Saline Gabazine | * | $P_{adj}$ = 0.0335 |
| | Descriptive | Saline (*n* = 7 zebrafish): 28.86 ± 2.314 | | | | |

| | | | | | | |
|---|---|---|---|---|---|---|
| | | Nicotine (*n* = 6 zebrafish): 38.67 ± 2.642<br>Gabazine (*n* = 6 zebrafish): 37.17 ± 1.641 | | | | |
| **6f** | One-way ANOVA | $F_{(2, 16)}$ = 9.548,<br>*P* = 0.0019 | Dunnett's test | Saline<br>Nicotine | ** | $P_{adj}$ = 0.0011 |
| | | | | Saline<br>Gabazine | * | $P_{adj}$ = 0.0326 |
| | Descriptive | Saline (*n* = 7 zebrafish): 4.829 ± 0.541<br>Nicotine (*n* = 6 zebrafish): 9.5 ± 0.928<br>Gabazine (*n* = 6 zebrafish): 7.7 ± 0.869 | | | | |
| ***Supplementary Figures*** | | | | | | |
| **Suppl 1**<br>0 week | Unpaired *t*-test | t = 0.043, df = 23 (Two-tailed) | | Control<br>Training | ns | *P* = 0.9660 |
| | Descriptive | Control (*n* = 10 zebrafish): 16.92 ± 0.366<br>Training (*n* = 15 zebrafish): 16.94 ± 0.29 | | | | |
| **Suppl 1**<br>1 week | Unpaired *t*-test | t = 0.775, df = 23 (Two-tailed) | | Control<br>Training | ns | *P* = 0.4459 |
| | Descriptive | Control (*n* = 10 zebrafish): 16.95 ± 0.35<br>Training (*n* = 15 zebrafish): 17.31 ± 0.3 | | | | |
| **Suppl 1**<br>2 weeks | Unpaired *t*-test | t = 2.193, df = 23 (Two-tailed) | | Control<br>Training | * | *P* = 0.0387 |
| | Descriptive | Control (*n* = 10 zebrafish): 17.01 ± 0.354<br>Training (*n* = 15 zebrafish): 18.06 ± 0.311 | | | | |
| **Suppl 1**<br>3 weeks | Unpaired *t*-test | t = 2.955, df = 23 (Two-tailed) | | Control<br>Training | ** | *P* = 0.0071 |
| | Descriptive | Control (*n* = 10 zebrafish): 17.14 ± 0.34<br>Training (*n* = 15 zebrafish): 18.66 ± 0.352 | | | | |
| **Suppl 1**<br>4 weeks | Unpaired *t*-test | t = 3.863, df = 23 (Two-tailed) | | Control<br>Training | *** | *P* = 0.0008 |
| | Descriptive | Control (*n* = 10 zebrafish): 17.21± 0.34<br>Training (*n* = 15 zebrafish): 19.53 ± 0.4336 | | | | |
| **Suppl 1**<br>5 weeks | Unpaired *t*-test | t = 4.65, df = 23 (Two-tailed) | | Control<br>Training | *** | *P* = 0.0001 |
| | Descriptive | Control (*n* = 10 zebrafish): 17.39 ± 0.335<br>Training (*n* = 15 zebrafish): 20.61 ± 0.517 | | | | |
| **Suppl 1**<br>6 weeks | Unpaired *t*-test | t = 5.244, df = 23 (Two-tailed) | | Control<br>Training | **** | *P* < 0.0001 |
| | Descriptive | Control (*n* = 10 zebrafish): 17.91 ± 0.33<br>Training (*n* = 15 zebrafish): 22.04 ± 0.6 | | | | |
| **Suppl 3b**<br>Mef-2 | Unpaired *t*-test | t = 0.243, df = 12 (Two-tailed) | | Control<br>Training | ns | *P* = 0.811 |
| | Descriptive | Control (*n* = 7 zebrafish): 66.53 ± 5.64<br>Training (*n* = 7 zebrafish): 68.49 ± 5.719 | | | | |
| **Suppl 3b**<br>HuC/D | Unpaired *t*-test | t = 1.751, df = 12 (Two-tailed) | | Control<br>Training | ns | *P* = 0.105 |
| | Descriptive | Control (*n* = 7 zebrafish): 53.77 ± 2.453<br>Training (*n* = 7 zebrafish): 64.36 ± 5.525 | | | | |
| **Suppl 3b**<br>NeuN | Unpaired *t*-test | t = 0.6, df = 12 (Two-tailed) | | Control<br>Training | ns | *P* = 0.559 |
| | Descriptive | Control (*n* = 7 zebrafish): 38.73 ± 3.763<br>Training (*n* = 7 zebrafish): 43.19 ± 6.405 | | | | |
| **Suppl 3b**<br>GFAP | Unpaired *t*-test | t = 0.391, df = 12 (Two-tailed) | | Control<br>Training | ns | *P* = 0.7 |
| | Descriptive | Control (*n* = 7 zebrafish): 20.94 ± 4.282<br>Training (*n* = 7 zebrafish): 18.66 ± 3.977 | | | | |
| **Suppl 4b** | Unpaired *t*-test | t = 8.226, df = 58 (Two-tailed) | | NSPCs<br>Neurons | **** | *P* < 0.0001 |
| | Descriptive | NSPCs (*n* = 12): -68.27 ± 0.749<br>Neurons (*n* = 48): -58.31 ± 0.573 | | | | |
| **Suppl 5c**<br>Frequency | One-way ANOVA | $F_{(2, 10)}$ = 9.036,<br>*P* = 0.0057 | Tukey's test | ACh (100μM)<br>ACh (5mM) | ** | $P_{adj}$ = 0.0095 |

| | | | | | | |
|---|---|---|---|---|---|---|
| | | | | ACh (100µM)<br>ACh (5mM) + TTX | ** | $P_{adj}$ = 0.0078 |
| | | | | ACh (5mM)<br>ACh (5mM) + TTX | ns | $P_{adj}$ = 0.88 |
| | Descriptive | ACh (100µM): 0.666 ± 0.064<br>ACh (5mM): 1.4 ± 0.133<br>ACh (5mM) + TTX: 1.486 ± 0.13 | | | | |
| **Suppl 5c**<br>Amplitude | One-way ANOVA | $F_{(2, 639)}$ = 0.111 | | | ns | *P* = 0.895 |
| | Descriptive | ACh (100µM): 21.22 ± 0.789<br>ACh (5mM): 21.48 ± 0.46<br>ACh (5mM) + TTX: 21.17 ± 0.493 | | | | |
| **Suppl 5d**<br>Amplitude | Unpaired *t*-test | t = 0.559, df = 13 (Two-tailed) | | GABA<br>GABA + TTX | ns | *P* = 0.5594 |
| | Descriptive | GABA (*n* = 11): 23.55 ± 1.524<br>GABA + TTX (*n* = 4): 21.82 ± 2.27 | | | | |
| **Suppl 5d**<br>Duration | Unpaired *t*-test | t = 1.351, df = 13 (Two-tailed) | | GABA<br>GABA + TTX | ns | *P* = 0.1999 |
| | Descriptive | GABA (*n* = 11): 72.73 ± 5.233<br>GABA + TTX (*n* = 4): 85.23 ± 4.596 | | | | |
| **Suppl 6b** | Descriptive | (*n* = 9): 1.312 ± 0.122 | | | | |
| **Suppl 6c**<br>Duration | Unpaired *t*-test | t = 4.753, df = 11 (Two-tailed) | | Saline<br>Mephenesin | *** | *P* = 0.0006 |
| | Descriptive | Saline (*n* = 9): 15.44 ± 1.215<br>Mephenesin (*n* = 4): 6.375 ± 0.625 | | | | |
| **Suppl 6c**<br>Amplitude | Unpaired *t*-test | t = 1.784, df = 11 (Two-tailed) | | Saline<br>Mephenesin | ns | *P* = 0.1020 |
| | Descriptive | Saline (*n* = 9): 1.002 ± 0.035<br>Mephenesin (*n* = 4): 0.895 ± 0.041 | | | | |
| **Suppl 7c** | Descriptive | Success rate (*n* = 8): 7.952 ± 0.461 | | | | |
| **Suppl 8b**<br>Cholinergic | One-way ANOVA | $F_{(3, 31)}$ = 43.0,<br>*P* < 0.0001 | Dunnett's test | Control<br>ACh | *** | $P_{adj}$ = 0.0006 |
| | | | | Control<br>Muscarine | ns | $P_{adj}$ = 0.6646 |
| | | | | Control<br>Nicotine | **** | $P_{adj}$ < 0.0001 |
| | Descriptive | Control (*n* = 9 zebrafish): 15.33 ± 1.74<br>ACh (*n* = 9 zebrafish): 27.33 ± 2.593<br>Muscarine (*n* = 8 zebrafish): 18.13 ± 1.141<br>Nicotine (*n* = 9 zebrafish): 44.78 ± 2.216 | | | | |
| **Suppl 8b**<br>GABAergic | One-way ANOVA | $F_{(2, 26)}$ = 207.6,<br>*P* < 0.0001 | Dunnett's test | Control<br>GABA | ** | $P_{adj}$ = 0.0056 |
| | | | | Control<br>Gabazine | **** | $P_{adj}$ < 0.0001 |
| | Descriptive | Control (*n* = 9 zebrafish): 15.33 ± 1.74<br>GABA (*n* = 11 zebrafish): 7.455 ± 0.511<br>Gabazine (*n* = 9 zebrafish): 54.11 ± 2.622 | | | | |
| **Suppl 9b**<br>With TTX | One-way ANOVA | $F_{(3, 18)}$ = 26.12,<br>*P* < 0.0001 | Dunnett's test | Control<br>Nicotine | * | $P_{adj}$ = 0.0176 |
| | | | | Control<br>GABA | ** | $P_{adj}$ = 0.0097 |
| | | | | Control<br>Gabazine | ** | $P_{adj}$ = 0.0035 |
| | Descriptive | Control (*n* = 4 zebrafish): 19.0 ± 1.78<br>Nicotine (*n* = 6 zebrafish): 29.83 ± 3.114<br>GABA (*n* = 6 zebrafish): 7.167 ± 0.98<br>Gabazine (*n* = 6 zebrafish): 32.5 ± 2.513 | | | | |

**Supplementary Table 2. Antibodies Used**[1]

| Antigen | Host | Source | Code | Dilution |
|---|---|---|---|---|
| ***Primary*** | | | | |
| BrdU | Mouse | Becton Dickinson | 347580; RRID: AB_10015219 | 1:80-1:100 |
| ChAT | Goat | Millipore | AB144P; RRID: AB_2079751 | 1:200 |
| Calbindin D-28K | Mouse | Swant | 300; RRID: AB_2079751 | 1:400-1:1000 |
| GABA | Rabbit | Sigma | A2052; RRID: AB_477652 | 1:2000 |
| GFAP | Rabbit | Cell Signaling | 12389; RRID: AB_2631098 | 1:200 |
| GFP | Rabbit | Molecular Probes | A-11122; RRID: AB_221569 | 1:500 |
| GFP | Chicken | Abcam | AB13970; RRID: AB_300798 | 1:600 |
| HuC/D | Mouse | Molecular Probes | A-21271; RRID: AB_221448 | 1:100 |
| HuC/D | Rabbit | GeneTex | GTX128365; RRID: N/A | 1:500 |
| Mef-2 | Rabbit | Santa Cruz | SC313; RRID: AB_631920 | 1:50-1:80 |
| NeuN | Rabbit | Cell Signaling | 24307; RRID: AB_2651140 | 1:500 |
| Sox2 | Goat | R&D Systems | AF2018; RRID: AB_355110 | 1:500 |
| ***Secondary*** | | | | |
| Goat IgG-568 | Donkey | ThermoFisher | A-11057; RRID: AB_2534104 | 1:500 |
| Goat IgG-488 | Donkey | ThermoFisher | A-11055; RRID: AB_2534102 | 1:500 |
| Chicken IgY-FITC | Donkey | ThermoFisher | SA1-72000; RRID: AB_923386 | 1:800 |
| Mouse IgG-647 | Donkey | ThermoFisher | A-31571; RRID: AB_162542 | 1:500 |
| Mouse IgG-568 | Donkey | ThermoFisher | A-10037; RRID: AB_2534013 | 1:500 |
| Mouse IgG-488 | Donkey | ThermoFisher | A-21202; RRID: AB_141607 | 1:500 |
| Rabbit IgG-488 | Donkey | ThermoFisher | A-21206; RRID: AB_2535792 | 1:500 |
| Rabbit IgG-647 | Donkey | ThermoFisher | A-31573; RRID: AB_2536183 | 1:500 |
| Rabbit IgG-568 | Donkey | ThermoFisher | A-10042; RRID: AB_2534017 | 1:500 |

[1]BrdU, *5*-Bromo-*2*'-Deoxyuridine; ChAT, choline-acetyltransferase; mef-2, myocyte enhancer factor-2; GFAP, Glial fibrillary acidic protein; GABA, γ-aminobutyric acid; GFP, green fluorescent protein; PCNA, proliferating cell nuclear antigen; Sox2, sex determining region Y-box 2.
