# Supplementary material for: Locomotion dependent neuron-glia interactions control neurogenesis and regeneration in the adult zebrafish spinal cord
Source: Nat Commun. 2021 Aug 11;12:4857. doi: 10.1038/s41467-021-25052-1 (PMC8357999; doi:10.1038/s41467-021-25052-1)
Supplement: Supplementary file 2 — Reporting summary. [file 41467_2021_25052_MOESM2_ESM.pdf]

nature research

Corresponding author(s): Ampatzis Konstantinos

Last updated by author(s): Jul 13, 2021

Reporting Summary

Nature Research wishes to improve the reproducibility of the work that we publish. This form provides structure for consistency and transparency in reporting. For further information on Nature Research policies, see our Editorial Policies and the Editorial Policy Checklist.

Statistics

For all statistical analyses, confirm that the following items are present in the figure legend, table legend, main text, or Methods section.

n/a	Confirmed	
☐	☒	The exact sample size (n) for each experimental group/condition, given as a discrete number and unit of measurement
☐	☒	A statement on whether measurements were taken from distinct samples or whether the same sample was measured repeatedly
☐	☒	The statistical test(s) used AND whether they are one- or two-sided<br>*Only common tests should be described solely by name; describe more complex techniques in the Methods section.*
☒	☐	A description of all covariates tested
☒	☐	A description of any assumptions or corrections, such as tests of normality and adjustment for multiple comparisons
☐	☒	A full description of the statistical parameters including central tendency (e.g. means) or other basic estimates (e.g. regression coefficient) AND variation (e.g. standard deviation) or associated estimates of uncertainty (e.g. confidence intervals)
☐	☒	For null hypothesis testing, the test statistic (e.g. F, t, r) with confidence intervals, effect sizes, degrees of freedom and P value noted<br>*Give P values as exact values whenever suitable.*
☒	☐	For Bayesian analysis, information on the choice of priors and Markov chain Monte Carlo settings
☒	☐	For hierarchical and complex designs, identification of the appropriate level for tests and full reporting of outcomes
☒	☐	Estimates of effect sizes (e.g. Cohen's d, Pearson's r), indicating how they were calculated

Our web collection on statistics for biologists contains articles on many of the points above.

Software and code

Policy information about availability of computer code

Data collection	All electrophysiology data were collected using pCLAMP 11 software suite (Clampex; Molecular Devices). All confocal images were acquired using the ZEN software (ZEISS).
Data analysis	Electrophysiology data analyzed using the pCLAMP 11 software suite (Clampfit v11.0; Molecular Devices) and AxoGraph (version X 1.5.4; AxoGraph Scientific, Sydney, Australia; RRID: SCR_014284).<br>Confocal microscopy image acquisition with ZEN (version 3.4/blue edition)<br>Anatomy data were analyzed using ImageJ/Fiji software (version 2.1.0/1.53c; https://imagej.nih.gov/ij/) and Origin 8 (OriginLab, Northampton, MA, USA).<br>Statistical analysis performed using Prism 9.0 (GraphPad Software Inc).<br>All images and figures processed using Photoshop (version 21.0.0) and Illustrator (version 24.1; Adobe Systems Inc., San Jose, CA, USA)

For manuscripts utilizing custom algorithms or software that are central to the research but not yet described in published literature, software must be made available to editors and reviewers. We strongly encourage code deposition in a community repository (e.g. GitHub). See the Nature Research guidelines for submitting code & software for further information.

Data

Policy information about availability of data

All manuscripts must include a data availability statement. This statement should provide the following information, where applicable:

- Accession codes, unique identifiers, or web links for publicly available datasets
- A list of figures that have associated raw data
- A description of any restrictions on data availability

All raw data used in this study to generate the figures and alyses are available as a source data file (.xls) as stated in the "Data availability" section. No custom or

open-source code was used for data collection and analyses.

Field-specific reporting

Please select the one below that is the best fit for your research. If you are not sure, read the appropriate sections before making your selection.

- [x] Life sciences
- [ ] Behavioural & social sciences
- [ ] Ecological, evolutionary & environmental sciences

For a reference copy of the document with all sections, see nature.com/documents/nr-reporting-summary-flat.pdf

Life sciences study design

All studies must disclose on these points even when the disclosure is negative.

Sample size

No statistical method was used to predetermine sample size. Sample sizes were chosen based on published studies and reported power analyses in the field, and are comparable to those in our previous publications (Chang, W., Pedroni, A., Hohendorf, V., Giacomello, S., Hibi, M., Köster, R.W., and Ampatzis, K. (2020). Functionally distinct Purkinje cell types show temporal precision in encoding locomotion. Proc. Natl. Acad. Sci. U.S.a. 117, 17330–17337; Pedroni, A., and Ampatzis, K. (2019). Large-Scale Analysis of the Diversity and Complexity of the Adult Spinal Cord Neurotransmitter Typology. iScience 19, 1189–1201; Bertuzzi, M., Chang, W., and Ampatzis, K. (2018). Adult spinal motoneurons change their neurotransmitter phenotype to control locomotion. Proc. Natl. Acad. Sci. U.S.a. 115, E9926–E9933; Bertuzzi, M., and Ampatzis, K. (2018). Spinal cholinergic interneurons differentially control motoneuron excitability and alter the locomotor network operational range. Sci Rep 8, 1988.).

Generally, sample sizes are based on the smallest number required to reach statistical significance in order to reduce unnecessary animal use.

Data exclusions

Zebrafish showing incorrect locomotor behavior and posture, and signs of significant distress after the in-vivo injection of drugs were excluded from analysis. Relevant experiments were few animals excluded from the analysis presented in the fig. 5b, 5c, 6d and 6e.

Replication

The number of independently replicated experiments is described in the figure legends and text. All replication attempts were successful.

Randomization

Zebrafish were randomly allocated into different experimental groups. No specific randomization method was used. Age/size-matched animals were used as controls in all experiments.

Blinding

The quantification of BrdU positive cells after training did not include a blind approach because the experiments performed in a stepwise manner. All manual counts of BrdU positive cells after drug administration were performed in a manner where the experimenters were blind to the conditions. In all quantifications multiple investigators participated independently to ensure the reproducibility of the data.
The experimenters were not blind to the experimental groups in the case of all electrophysiological recordings and behavioral experiments as the investigators perform visual guided recordings of selective cells (V2a-INs and NSPCs) that are expressing the green fluorescent protein GFP. Regarding the behavioral experiments the investigators performed the experiments in sequential manner (one group at a time), thus, blinding was not applicable.

Reporting for specific materials, systems and methods

We require information from authors about some types of materials, experimental systems and methods used in many studies. Here, indicate whether each material, system or method listed is relevant to your study. If you are not sure if a list item applies to your research, read the appropriate section before selecting a response.

Materials & experimental systems

n/a	Involved in the study	
[ ]	[x]	Antibodies
[x]	[ ]	Eukaryotic cell lines
[x]	[ ]	Palaeontology and archaeology
[ ]	[x]	Animals and other organisms
[x]	[ ]	Human research participants
[x]	[ ]	Clinical data
[x]	[ ]	Dual use research of concern

Methods

n/a	Involved in the study	
[x]	[ ]	ChIP-seq
[x]	[ ]	Flow cytometry
[x]	[ ]	MRI-based neuroimaging

Antibodies

Antibodies used

All antibodies (primary and secondary) used in this study, along with details about the species raised, dilution used, and RRID numbers, are provided in great detail in Supplementary Table 2.

Validation

All antibodies have been validated extensively in numerous previous publications:

BrdU: widely used marker for detection of the exogenous epitope of BrdU. The manufacturer validated the antibody (https://

www.bdbiosciences.com/us/applications/research/apoptosis/purified-antibodies/purified-mouse-anti-brdu-b44/p/347580). Moreover, the antibody ability to label the BrdU positive cells in zebrafish was based on our previously published results (e.g., Ampatzis , and Dermon (2007). Eur. J. Neurosci. 25, 1030–1040; Ampatzis, et al., (2012). Neuroscience 226, 367–381) and from others (Caron et al., 2008, Development; McGraw et al., 2008, Journal of Neuroscience).
ChAT: widely used marker for detection of cholinergic neurons in numerous species with over 270 citations. The antibody was extensively used before in zebrafish from our lab and others (Pedroni and Ampatzis, 2019. iScience; Berg et al., 2018. Brain Struct Funct; Bertuzzi and Ampatzis, 2018. Sci Rep; DeMarko et al., 2019. Journal of Comparative Neurology; DeOliveira-Mello, 2019. Brain Res.) with 45 zebrafish references (http://zfin.org/ZDB-ATB-081017-3#summary).
Calbindin D-28K: Common used antibody for the detection of calcium binding protein Calbindin D-28K. The antibody is used before in zebrafish (Berg, E.M., Bertuzzi, M., and Ampatzis, K. 2018. Brain Struct Funct 223, 2181–2196.; Mateos, J.M., Barmettler, G., Doehner, J. et al. 2018. Sci Rep 8, 11610; Trotha, von, J.W., Vernier, P., and Bally-Cuif, L. 2014. Eur. J. Neurosci. 40, 3302–3315; Lindsey, B.W., Darabie, A., and Tropepe, V. 2012. J. Comp. Neurol. 520, 2275–2316; Zupanc, G.K.H., Hinsch, K., and Gage, F.H. 2005. J. Comp. Neurol. 488, 290–319; Namikawa, K., Dorigo, A., Zagrebelsky, M., Russo, G., Kirmann, T., Fahr, W., Dübel, S., Korte, M., and Köster, R.W. 2019. J. Neurosci. 39, 3948–3969.) and in other fish species (Graña, P., Folgueira, M., Huesa, G., Anadón, R., and Yáñez, J. 2013.J. Comp. Neurol. 521, 2454–2485) nervous system.
GABA: widely used marker for detection of GABAergic neurons with 147 references (https://scicrunch.org/resources/Any/search?q=AB_477652&l=AB_477652). In zebrafish, the field is extensively used to detect GABAergic neurons in both the brain and spinal cord from our lab (Pedroni and Ampatzis, 2019. iScience; Berg et al., 2018. Brain Struct Funct) and others with 38 references (http://zfin.org/ZDB-ATB-081106-2#summary).
GFAP: widely used marker for detection of astrocytes in multiple animal models with more than ten references.
GFP (chicken): It is a widely used marker for detecting GFP in several models, including the zebrafish with more than 490 citations including zebrafish (Hall et al., 2009, Journal of Leukocyte Biology; Lee at al., 2013, Development; Groteck et al., 2013, Development).
GFP (rabbit): It is a widely used marker for detecting GFP in several models, including the zebrafish with more than 330 citations. The antibody was used before in zebrafish (http://zfin.org/ZDB-ATB-081009-2#summary).
HuC/D (mouse): A widely used marker for detecting eval3&4 neuronal proteins and therefore an accurate neuronal marker. The antibody has 390 citations in zebrafish (http://zfin.org/ZDB-ATB-081003-2#summary) and another 300 citations in other animals.
HuC/D (rabbit): A zebrafish specific antibody against the neuronal proteins Elav3+4 (https://www.genetex.com/Product/Detail/Elavl3-4-antibody/GTX128365). As such, the manufacturer validated the antibody. The antibody used before in zebrafish spinal cord (Pedroni and Ampatzis, 2019. iScience; Berg et al., 2018. Brain Struct Funct; Li et al., 2019. Stem cells research & therapy).
Mef-2: The antibody used extensively in zebrafish research with 57 citations (http://zfin.org/ZDB-ATB-090826-2#summary).
NeuN: antibody widely used as a neuronal marker with more than 40 citations (https://www.cellsignal.com/products/primary-antibodies/neun-d4g4o-xp-rabbit-mab/24307). Moreover, the manufacturer validated the antibody by WB.
Sox2: Antibody used before 91 citations to detect SOX2 belongs to the SOX (SRY-like HMG box) family of transcription factors commonly used as a marker of the stem cells. The manufacturer validated the antibody (https://www.rndsystems.com/products/human-mouse-rat-sox2-antibody_af2018) and was used before in zebrafish research (Leichsenring et al., 2013. Science; Berg et al., 2018. Brain Struct Funct; Iribarne et al., 2019. Frontiers in cell and developmental biology; Gorsuch et al., 2017. Experimental Eye Research; Kamachi et al., 2008. Genesis).

Animals and other organisms

Policy information about studies involving animals; ARRIVE guidelines recommended for reporting animal research

Laboratory animals	Only juvenile/adult zebrafish (8-10 weeks post fertilization) of both sexes were used in this study.<br>Wild type: AB/Tübingen<br>Transgenic animals: Tg(Chx10:GFPnns1) and Tg(her4.1:GFP)<br>More details regarding the age, size, sex and number used in this study are provided in the Methods section.
Wild animals	This study did not involve wild animals.
Field-collected samples	This study did not involve samples collected from the field.
Ethics oversight	The local Animal Research Ethical Committee (at Karolinska Institutet) approved all experimental protocols, Stockholm (Ethical permit no. 9248-2017), and were implemented under EU guidelines for the care and use of laboratory animals (2010/63/EU).

Note that full information on the approval of the study protocol must also be provided in the manuscript.
